# A series of *tert*-butyl- and *tert*-butylthio-substituted phthalocyanine derivatives: biological activities, DFT calculations and molecular docking studies

**DOI:** 10.1039/d6ra02238c

**Published:** 2026-07-13

**Authors:** Gülşah Tollu, Gizem Gümüşgöz Çelik, Gülenay Tunç, Sadin Özdemir, Savaş Kaya, Avni Berisha, Ayşe Gül Gürek, Devrim Atilla

**Affiliations:** a Laboratory and Veterinary Health, Technical Science Vocational School, Mersin University TR-33343 Mersin Türkiye; b Department of Chemistry, Gebze Technical University 41400 Gebze Kocaeli Türkiye datilla@gtu.edu.tr +0090-262-6053005 +0090-262-6053020; c Food Processing Programme, Technical Science Vocational School, Mersin University Yenisehir 33343 Mersin Türkiye; d Sivas Cumhuriyet University, Faculty of Science, Department of Chemistry 58140 Sivas Türkiye; e Department of Chemistry, Faculty of Natural and Mathematics Science, University of Prishtina 10000 Prishtina Kosovo

## Abstract

In this study, the biological activities, including amylolytic, antimicrobial, antimicrobial photodynamic therapy (aPDT), antibiofilm, DNA cleavage, and microbial cell viability inhibition activities, of a series of *tert*-butyl-substituted phthalocyanines (*t*BuH_2_Pc, *t*BuZnPc, *t*BuNiPc, and *t*BuCuPc) and their *tert*-butylthio-substituted derivatives (S-*t*BuH_2_Pc, S-*t*BuZnPc, S-*t*BuNiPc, and S-*t*BuCuPc) were systematically evaluated and compared. In addition, density functional theory (DFT) calculations and molecular docking studies targeting α-amylase were conducted for all the compounds. All of the phthalocyanines (Pcs) demonstrated effective amylolytic inhibition activity, especially at 50 and 100 mg L^−1^, indicating that the test compounds may be effective antidiabetic agents. Among the tested molecules, S-*t*BuZnPc and S-*t*BuCuPc exhibited superior antibiofilm activity against *S. aureus* and *P. aeruginosa*, achieving complete inhibition at 15 mg L^−1^ under both dark and LED light conditions. DNA cleavage experiments showed that all the Pc derivatives except *t*BuH_2_Pc and *t*BuNiPc induced complete cleavage at all tested concentrations. Microbial viability assays revealed that *t*BuZnPc, *t*BuCuPc, S-*t*BuZnPc and S-*t*BuCuPc achieved 100% inhibition of *E. coli* viability at just 10 mg L^−1^. DFT calculations confirmed favorable electronic distributions and molecular electrostatic potential maps, while molecular docking results demonstrated strong binding affinities of the S-*t*BuPc compounds to α-amylase, indicating their potential biological relevance. Collectively, the incorporation of S-*t*Bu groups into the Pc macrocycle significantly enhanced both biological and photodynamic efficacies, underscoring the biomedical potential of these Pc derivatives.

## Abstract 2

The biological activities of a series of *tert*-butyl-substituted Pcs and their *tert*-butyl thio-substituted derivatives were evaluated and compared. DFT calculations and molecular docking studies targeting α-amylase were conducted for all the Pcs.

## Graphical abstract

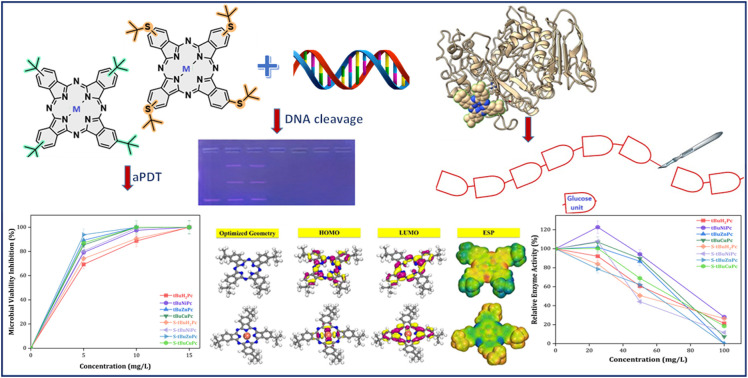

## 1. Introduction

Phthalocyanine (Pc) macrocycles, which consist of a conjugated aromatic system, exhibit remarkable thermal and chemical stability, as well as tunable photo-physicochemical and electrochemical properties.^1^ These characteristics largely depend on the nature of the metal ion coordinated to the central cavity of the Pc core and the type and position (peripheral, non-peripheral, or axial) of the substituent attached to the macrocycle.^2^ Owing to their structural versatility, Pc derivatives provide fine-tuning properties, such as solubility and biological activity, to the macrocycle, thereby making it available for various scientific and technological applications.^3^

The biological activities and applications of Pc derivatives, including antimicrobial activity,^4,5^ DNA cleavage,^2,4,5^ enzyme inhibition,^6^ and antimicrobial photodynamic therapy (aPDT),^2,7–11^ have been extensively studied. The growing interest in Pc macrocycles for biological applications is largely attributed to their structural flexibility, which allows for the introduction of activity-enhancing substituents and/or coordination with metal ions that improve their biological activity.

Molecular docking is a powerful computational approach used to explore how small molecules, such as potential drug candidates, might interact with a target protein. By simulating the way a ligand fits into the active site of a protein, docking helps to predict not only how strongly the two might bind but also how they orient and interact at the molecular level. This method has become a cornerstone of modern drug discovery, offering a fast and cost-effective way to screen compounds, understand binding mechanisms, and guide the design of more effective therapeutics. Through energy-based scoring, docking evaluates multiple possible binding poses, considering factors like shape complementarity, hydrogen bonding, and electrostatic attraction, ultimately shedding light on the molecular interactions that drive biological activity.^12–17^

Numerous studies reported in the literature have focused on *tert*-butyl-substituted Pc derivatives for various applications, such as solar cell fabrication,^18,19^ Langmuir–Blodgett film preparation,^20^ electrochemistry,^21^ sensing,^22–24^ bioimaging,^25^ and PDT.^26,27^ However, there is currently no comprehensive and systematic study addressing the biological activity of these compounds in relation to different central metal ions and substituent types (*tert*-butyl *versus tert*-butylthio). To the best of our knowledge, this is the first systematic study to address this gap, highlighting the novelty of our work.

**Fig. 1:**
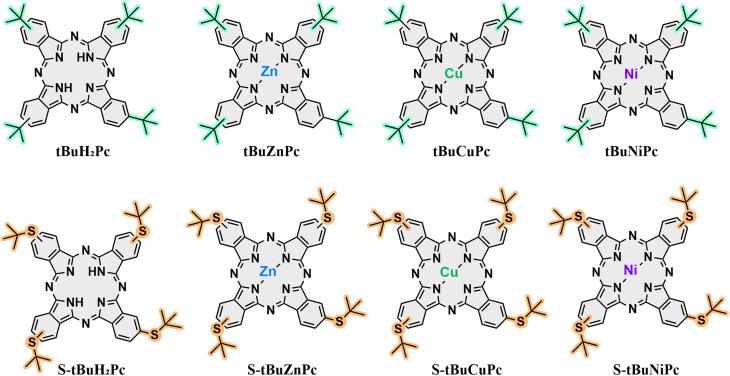
Molecular structures of the investigated compounds.

The *tert-*butyl group is strategically utilized to enhance the solubility of the Pc macrocycle^28^ and to suppress aggregation through steric hindrance around the macrocyclic core.^29^ This study aims to systematically evaluate how the nature of the peripheral substituents (*tert-*butyl and *tert*-butylthio groups) and the central metal ions influence the biological performance of *t*BuH_2_Pc, *t*BuZnPc, *t*BuNiPc, and *t*BuCuPc, along with their thio-substituted counterparts S-*t*BuH_2_Pc, S-*t*BuZnPc, S-*t*BuNiPc, and S-*t*BuCuPc. Particular focus is placed on assessing their antimicrobial efficacy, aPDT potential, α-amylase inhibition, DNA cleavage efficiency, and antibiofilm activity. The molecular structures of all the investigated MPcs are illustrated in Fig. 1. Furthermore, density functional theory (DFT) calculations and molecular docking simulations against α-amylase were conducted for all the Pc derivatives to complement the experimental findings.

## 2. Experimental

### 2.1. Instruments and materials

All of the chemicals and solvents were obtained from commercial suppliers and used without further purification. Metal salts (chloride or acetate forms, anhydrous, ≥99%, Merck) were used as metal sources in the syntheses. Silica gel 60 F_254_ on aluminum sheets (E. Merck) was used for thin-layer chromatography (TLC), while silica gel (230–400 mesh, 0.040–0.063 mm, Merck) was employed for column chromatography. Dithranol (DIT, ≥98%, Sigma-Aldrich) served as the MALDI-TOF matrix. All instrumental measurements were carried out using standard laboratory equipment. Elemental analysis was performed using a Thermo Finnigan Flash 1112 instrument, while additional analyses were performed using a Carlo Erba 1106 elemental analyzer. Infrared spectra were recorded using a PerkinElmer FT-IR spectrometer equipped with an ATR accessory containing a zinc selenide (ZnSe) crystal. ^1^H NMR spectra were obtained using Bruker 500 MHz and Varian INNOVA 500 MHz spectrometers. Matrix-assisted laser desorption/ionization time-of-flight mass spectrometry (MALDI-TOF-MS) measurements were carried out using a Bruker Daltonics MicrOTOF. Electronic absorption spectra were recorded using a Shimadzu UV-2600 UV-vis spectrophotometer, and the steady-state fluorescence spectra of solutions were recorded using a Cary Eclipse spectrofluorometer (Varian, USA).

### 2.2. Synthesis

*t*BuH_2_Pc, *t*BuZnPc, *t*BuNiPc, and *t*BuCuPc were synthesized using slightly modified versions of reported procedures,^30–33^ whereas S-*t*BuH_2_Pc, S-*t*BuZnPc, and S-*t*BuCuPc were synthesized according to a previously reported method.^34^

#### 2.2.1. 2(3),9(10),16(17),23(24)-Tetrakis(*tert*-butylsulfanyl)phthalocyaninatonickel(ii) (S-*t*BuNiPc)

A mixture of 4-(*tert*-butylthio)phthalonitrile (1) (0.216 g, 1 mmol), NiCl_2_ (0.259 g, 2 mmol), and a catalytic amount of 1,8-diazabicyclo[5.4.0]undec-7-ene (DBU) in *n*-pentanol (1 mL) was heated under argon with continuous stirring for 24 h. After cooling to room temperature, the reaction mixture was precipitated by the addition of ethanol. The resulting solid was collected by filtration, washed several times with ethanol, and dried. The bluish crude product was further purified by column chromatography on silica gel using DCM/ethanol (100 : 5, v/v) as the eluent. Yield: 67 mg (27%). FT-IR: (cm^−1^): 3084–3000 (Ar–CH), 2960–2860 (Aliph-CH), 1604 (–C=N–). ^1^H NMR (CDCl_3_): *δ*, ppm 8.10–7.46 (Ar–H, m, 12H), 1.51(–CH_3_, m, 36H). UV-vis (THF) *λ*_max_/nm: 335, 673. MALDI-TOF-MS *m*/*z* (C_48_H_48_N_8_S_4_Ni) found = 923.31 (Calcd. for [M]^+^ 923.90).

### 2.3. Biological activity test

#### 2.3.1 α-Amylase activity

All Pc derivatives were transferred into test tubes at concentrations of 25–100 mg L^−1^, followed by the addition of phosphate buffer and α-amylase. The mixtures were incubated at 37 °C for 15 minutes. Then, 0.2 mL of 1% potato starch solution was added to initiate the hydrolysis process, and the mixtures were incubated at 37 °C for an additional 20 minutes. After that, 0.4 mL of 3.5 dinitrosalicylic acid (DNS) was added to each test tube to stop the hydrolysis process, and the tubes were kept in boiling water for 5 minutes. After cooling, the mixtures were diluted with 3 mL of distilled water, and spectrophotometric readings were taken at 540 nm. A solution without a test sample served as the control. All results were then evaluated using eqn (1) as follows:^35^

1 Amylolytic activity (%) = 100 − (Control_abs_ − Sample_abs_)/Control_abs_ × 100.

#### 2.3.2. DNA cleavage activity

**Scheme 1:**
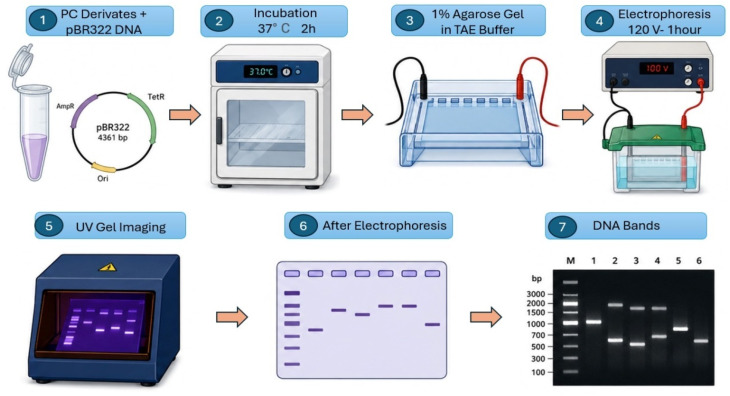
Schematic of the procedure of the interactions of the Pc derivatives with pBR322 plasmid DNA by an agarose gel electrophoresis assay.

The cleavage activity of Pc derivatives with plasmid pBR322 DNA was investigated using an agarose gel electrophoresis process. Briefly, reaction mixtures were prepared by mixing 15 µL of the Pc derivatives with 5 µL of pBR322 plasmid DNA in PCR tubes. The mixtures were then incubated at 37 °C for 2 h under dark conditions. A 1% (w/v) agarose gel was prepared and placed in an electrophoresis chamber containing Tris-acetate-EDTA (TAE) buffer. After incubation, each reaction mixture was blended with 3 µL of loading dye prior to being carefully introduced into the gel wells, and then, electrophoresis was performed at 120 V for 1 hour. The gel was then gently removed from the electrophoresis tank, and finally, the DNA bands were visualized using a UV transilluminator to visualize the gel.^36^ The schematic of the procedure of the interactions of PC derivatives with pBR322 plasmid DNA by an agarose gel electrophoresis assay is presented in Scheme 1.

#### 2.3.3. Antimicrobial activity

The microdilution method mentioned in the reference was used to evaluate the antimicrobial activities of Pcs.^37^ The microorganisms used in the experiment to determine antimicrobial activities were *Enterococcus hirae* (ATCC 10541), *Escherichia coli* (ATCC 10536), *Staphylococcus aureus* (ATCC 6538), *Enterococcus faecalis*, *Legionella pneumophila* subsp. *pneumophila* (ATCC 33152), *Pseudomonas aeruginosa* (ATCC 9027), *Candida parapsilosis,* and *Candida tropicalis*. To prepare the cultures, test microorganisms were grown in nutrient broth (NB) at 37 °C for 24 h before the assays. First, 150 µL of NB was added to 96-well plates, and two-fold serial dilutions of the Pc derivatives were performed. The wells were then inoculated with the microorganisms to achieve a concentration of 2.8 × 10^8^ CFU mL^−1^. After a 24-h incubation period at 37 °C, the minimum inhibitory concentration (MIC) values were determined based on the lowest concentration of the compounds that inhibited microbial growth.

#### 2.3.4. Antibiofilm and aPDT activity

*S. aureus* and *P. aeruginosa* were used as test microorganisms to determine the ability of Pcs to inhibit biofilm production. Nutrient broth (NB) media were first added to 24-well plates, and the samples prepared at different concentrations (62.5, 125, and 250 mg L^−1^) were added and inoculated separately into the 24-well plates. It was then incubated at 37 °C for three days to ensure cell adhesion to the surface. Following incubation, the biofilm-covered wells of the 24-well plate were carefully washed twice with 200 µL of phosphate-buffered saline (PBS) and allowed to dry in air for 30 min. After these procedures, an aqueous crystal violet solution (200 µL, 1%) was added to each well of the plate to determine the biofilm formation. The biofilm was allowed to stain for 60 min, and the plates were then washed using PBS to remove CV. After all these processes, ethanol was added and left at room temperature for 15 minutes to recover the absorbed CV. In addition, the same method was applied for biofilm inhibition activity with aPDT, but in the last stage before bacterial inoculation, the compounds were exposed to LED light for 30 minutes for the aPDT study.^38^ A red-orange light-emitting diode was used at 632 ± 2 nm with a dosage of 12 J^.^cm^2^. Biofilm inhibition and aPDT activities were calculated by a spectrophotometer at 595 nm using eqn (2) as follows:2
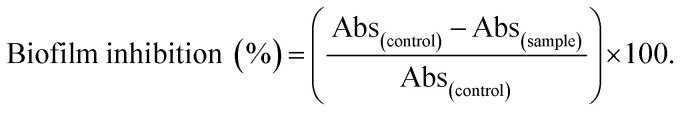


#### 2.3.5. Microbial cell viability inhibition

*E. coli* strain (ATCC 25922) was used as the microbial species to investigate the microbial cell viability of the Pcs. *E. coli* was first revived before the study began. It was then inoculated into a nutrient broth (NB) medium and cultured for one day at 37.5 °C at 150 rpm in a shaker. It was then centrifuged and washed twice with sterile distilled water. After these steps, *E. coli* was suspended in 10 mL of saline (0.9%). The microbial cell viability assay was performed using this suspension (2.8 × 10^9^ CFU mL^−1^). *E. coli* was treated with the Pcs at levels of 5–10 mg L^−1^ at 37 °C for 2 h. After incubation, the mixtures were diluted in different ratios and inoculated into a nutrient agar medium. All the samples were incubated at 37.5 °C for 24 hours. After all these procedures, colonies were counted. Applications without the Pcs were used as controls. The following equation (*x*) was used to evaluate the microbial cell viability inhibition, and the calculations were carried out with reference to eqn (3) as follows:^37^

3 Microbial cell viability inhibition (%) = [(*A*_control_ − *A*_sample_/*A*_control_)] × 100.

### 2.4. Theoretical calculations and methodology

#### 2.4.1. Details of density functional theory calculations

All calculations were carried out using density functional theory (DFT) with the M06-L *meta*-GGA exchange–correlation functional and a DND basis set.^39^ No pseudopotentials were applied. The electronic structure was treated using an unrestricted spin-polarized approach with zero net charge. Geometry optimizations were performed with the following convergence criteria: energy change below 1.0 × 10^−5^ Ha, and maximum displacement below 5.0 × 10^−3^ Å, with a limit of 999 optimization steps and a maximum displacement of 0.3 Å per step. An improved initial Hessian was employed, and symmetry constraints were disabled. The SCF procedure used Pulay's DIIS extrapolation, a maximum of 9999 iterations with a density convergence threshold of 1.0 × 10^−5^. Solvent effects were considered using the COSMO model^40^ with water as the solvent (dielectric constant *ε* = 78.54). The global cutoff radius for numerical integration was set to 3.3 Å.

For the mathematical definitions of the chemical reactivity descriptors, such as chemical potential (*µ*), electronegativity (*χ*), hardness (*η*), and softness (*σ*), conceptual density functional theory (CDFT) presents the following eqn (4)–(6):^41,42^4
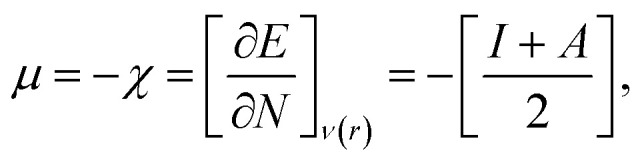
5
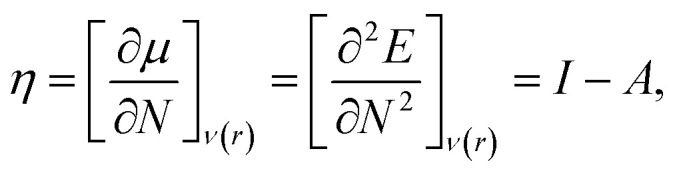

6 *σ* = 1/*η*.

In the given equations, *E* and N stand for the total electronic energy and total number of the electrons of the chemical system, respectively. *I* and *A* are the ionization energy and electron affinity, respectively. For the approximately prediction of the ionization energy and electron affinity of the molecules mentioned in this paper, Koopmans' Theorem (KT),^43^ known with the following HOMO and LUMO orbital energy bases relations was taken into account:

7 *I* = −*E*_HOMO_,

8 *A* = −*E*_LUMO_.

One of the parameters commonly used in stability analysis is the electrophilicity index because this parameter is frequently used, especially by organic chemists, to discuss and compare the electron-donating abilities of chemical systems.

Although many electrophilicity scales have been developed, two commonly used electrophilicity indexes, *ω*_1_ and *ω*_2_, are calculated as follows:^44,45^

9 *ω*_1_ = *χ*^2^/2*η* = *µ*^2^/2*η*,

10
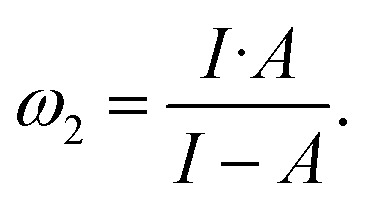
In the present paper, the chemical reactivities of the synthesized chemical systems will be examined and compared using CDFT^46^-based electronic structure principles. Then, biological activities determined through experiments and Molecular Docking analysis of the compounds will be explained using CDFT descriptors.

#### 2.4.2. Molecular docking analyses

Molecular docking is a powerful computational approach used to explore how small molecules, such as potential drug candidates, might interact with a target protein. By simulating the way a ligand fits into the protein's active site, docking helps predict not only how strongly the two might bind, but also how they orient and interact at the molecular level. This method has become a cornerstone of modern drug discovery, offering a fast and cost-effective way to screen compounds, understand binding mechanisms, and guide the design of more effective therapeutics. Through energy-based scoring, docking evaluates multiple possible binding poses, considering factors like shape complementarity, hydrogen bonding, and electrostatic attraction, ultimately shedding light on the molecular interactions that drive biological activity.^17,42,47–50^

#### 2.4.3. Molecular docking protocol

Molecular docking studies were conducted using Molegro Virtual Docker (MVD),^51^ a structure-based drug design tool that enables accurate prediction of ligand–protein interactions by simulating the preferred orientation and binding conformation of small molecules within a macromolecular target. The crystal structure of human pancreatic α-amylase (PDB ID: 1HNY)^52^ was obtained from the RCSB Protein Data Bank^53^ and prepared by removing water molecules, adding hydrogen atoms, and assigning appropriate protonation states under physiological pH. Ligand structures, including *t*-butyl-substituted phthalocyanines with Cu(ii), Zn(ii), Ni(ii), and metal-free cores, were constructed using molecular modeling software and energy-minimized prior to docking. All docking simulations were performed within the Molegro Docking Wizard, using the MolDock scoring function, which is based on a piecewise linear potential (PLP) energy model that combines van der Waals, electrostatic, hydrogen bonding, and internal strain components. The search space was defined by centering a sphere of 15 Å radius around the enzyme's active site, with the grid resolution set to 0.3 Å. The MolDock SE (Simplex Evolution) algorithm was employed for pose generation, with parameters set as follows: population size = 50, maximum iterations = 1500, and energy evaluations = 300 000. Ten docking runs were performed for each ligand, and the pose with the lowest MolDock score was selected for further analysis.

## 3. Results and discussion

### 3.1. Synthesis

**Scheme 2:**
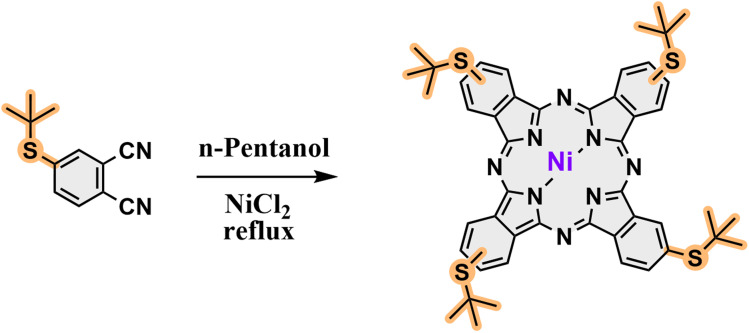
Reaction scheme of S-*t*BuNiPc from the *tert*-butyl-thio-substituted phthalonitrile.

Since *t*BuH_2_Pc, *t*BuZnPc, *t*BuNiPc, and *t*BuCuPc are commonly studied Pc derivatives, their synthesis using various methods, including microwave-assisted synthesis, has been well documented in the literature.^30–33^ However, within the scope of this study, these compounds were synthesized using slightly modified procedures based on existing methods. The detailed synthesis methods and characterization of these compounds are provided in the SI file. S-*t*BuH_2_Pc, S-*t*BuZnPc, and S-*t*BuCuPc were synthesized following the procedures reported in the literature,^34^ while S-*t*BuNiPc was newly prepared using a modified method described in this work, as illustrated in Scheme 2.

In this study, S-*t*BuNiPc was synthesized by reacting with the corresponding *tert*-butyl-thio-substituted phthalonitrile with a nickel(ii) salt in the presence of DBU under reflux in *n*-pentanol for 24 hours (Scheme 2). The reaction afforded the desired complex in a comparable yield of 24%. The synthesized Pc was fully characterized by FT-IR spectroscopy, ^1^H NMR spectroscopy, and mass spectrometry, along with elemental analyses (Fig. S6–S8). The results of these analyses confirmed the proposed molecular structure and high purity of S-*t*BuNiPc.

### 3.2. Photophysical properties of Pc derivatives

**Fig. 2:**
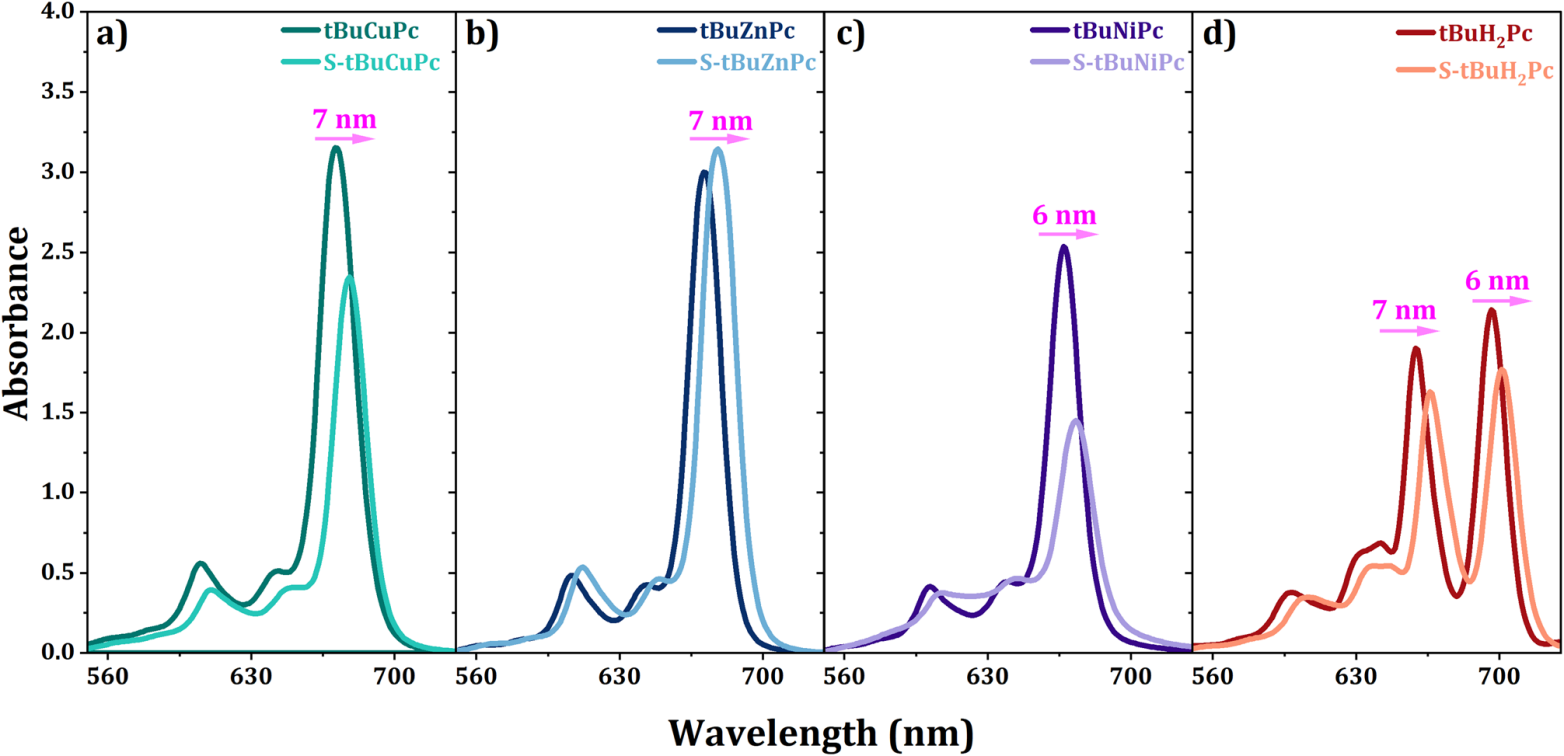
UV-vis absorption spectra of the *tert*-butyl substituted phthalocyanines (*t*BuMPc) and their thio-substituted counterparts (S-*t*BuMPc): (a) *t*BuCuPc and S-*t*BuCuPc, (b) *t*BuZnPc and S-*t*BuZnPc, (c) *t*BuNiPc and S-*t*BuNiPc, and (d) *t*BuH_2_Pc and S-*t*BuH_2_Pc. All spectra were recorded in THF at a constant concentration (12 µM) using a 1 cm quartz cuvette at room temperature.

The photophysical properties of the *t*BuPcs and S-*t*BuPcs derivatives were investigated in THF, which provided the best solubility for all compounds. This study aimed to elucidate the electronic and steric effects of the central metal ions (H_2_, Zn^2+^, Cu^2+^, and Ni^2+^) and the sulfur donor atom. All the compounds exhibited absorption spectra with characteristic Soret (B-band) and Q-band regions typical of the Pc macrocycle. Metal-free derivatives (*D*_2h_ symmetry) exhibited a split Q-band, whereas metallated Pcs (*D*_4h_ symmetry) showed a single, sharp band because of orbital degeneracy removal upon metal coordination.^54^ The same spectral behavior was also observed in the present *t*BuPc and S-*t*BuPc series. The S-*t*BuPc series showed a consistent red shift of about 6–7 nm in the Q-band compared with the corresponding *t*BuPc derivatives. Such behavior arises from the electron-donating (p-donor) character of the thio group, which can interact with the π-system, lowering the HOMO–LUMO energy gap and resulting in absorption at longer wavelengths. This observation is consistent with the findings of Kobayashi *et al.*, who reported that thioalkyl substitution causes a bathochromic shift in the Q-band due to resonance between sulfur 3p and the π-system, leading to a narrower HOMO–LUMO gap.^55^ For a clearer comparison, the overlaid spectra are displayed in Fig. 2.

**Fig. 3:**
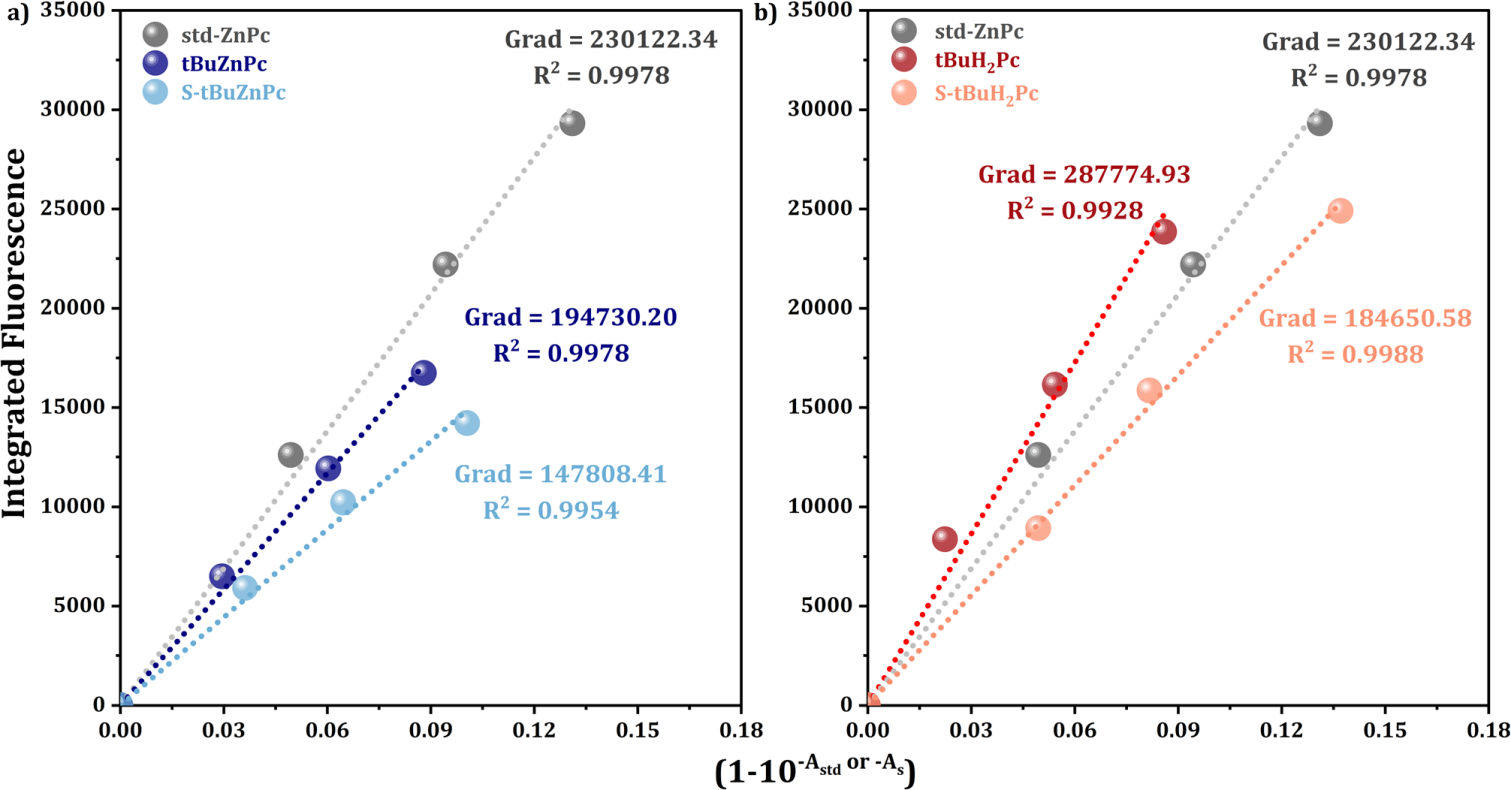
Integrated fluorescence intensity *vs.* 1 − 10^−*A*_std_ or −^*^A^*^_s_^ plots used for the quantum yield determination of (a) ZnPc derivatives and (b) H_2_Pc derivatives in THF. Measurements were performed at concentrations ranging from 0.25 to 1.50 µM (*λ*_ex_ = 635 nm, slit width = 5 nm, and at room temperature).

To determine the behavior in THF, the absorbance changes observed in the UV-vis spectra recorded at 2–12 µM within 300–800 nm were analyzed to evaluate the possible aggregation. The corresponding spectra are provided in the SI (Fig. S11–S18), and the comparative molar absorption coefficients (*ε*) are shown in Fig. 3. S-*t*BuNiPc and S-*t*BuZnPc exhibited a noticeable decrease in absorbance above 8 µM, while no significant change was observed for the others, which remained monomeric throughout this concentration range. A negative deviation from the linear trend was observed for S-*t*BuNiPc and S-*t*BuZnPc (red cross in Fig. S19), indicating a violation of the Beer–Lambert law and the onset of aggregation through π–π stacking at this critical concentration threshold. The molar absorption coefficients (log *ε*) of the compounds, presented in Table 1, indicate intense π–π* transitions typical of Pcs.

**Table 1:** Photophysical and photochemical parameters for MPc derivatives in THF

Compound	B Band λ^abs^_max_ (nm)	Q band λ^abs^_max_ (nm)	Q band log *ε*	λ^em^_max_ (nm)	*Φ*_F_
*t*BuCuPc	345	672	5.44	—	—
*t*BuZnPc	348	671	5.42	679	0.21
*t*BuNiPc	332	667	5.32	—	—
*t*BuH_2_Pc	341	660; 696	5.21; 5.26	702	0.31
S-*t*BuCuPc	350	679	5.29	—	—
S-*t*BuZnPc	355	678	5.48	687	0.16
S-*t*BuNiPc	335	673	5.14	—	—
S-*t*BuH_2_Pc	343	667; 702	5.14; 5.17	707	0.20
std-ZnPc		666	5.19	673	0.25

To investigate the fluorescence behavior and to determine the quantum yields (*Φ*_F_) of the compounds, emission spectra were recorded within the 0.25–1.5 µM concentration range using an excitation wavelength of 635 nm. The quantum yields were calculated by the gradient method, using unsubstituted ZnPc as a standard reference under the same conditions, and the detailed procedures are given in the SI. The recorded spectra are included in the SI (Fig. S20), and the comparative *Φ*_F_ plots are presented in Fig. 3. All photophysical parameters are summarized in Table 1. The *Φ*_F_ values are in the order of H_2_Pc (0.31) > ZnPc (0.25) > *t*BuZnPc (0.21) > S-*t*BuH_2_Pc (0.20) > S-*t*BuZnPc (0.16), while Cu(ii) and Ni(ii) complexes were non-emissive. This behavior is attributed to the efficient intersystem crossing (ISC) induced by the Cu(ii) and Ni(ii) centers, which promotes non-radiative S_1_ → T_1_ transitions. In contrast, the diamagnetic Zn(ii) and metal-free systems, having weaker spin–orbit coupling, favor radiative S_1_ → S_0_ relaxation, resulting in measurable fluorescence. The slightly higher *Φ*_F_ values of the H_2_Pc derivatives compared to their Zn(ii) analogues are consistent with the reduced spin–orbit interaction in the absence of a central metal atom, as reported previously.^28^ Within the metallated series, the introduction of thioalkyl (S-*t*Bu) groups caused a minor decrease in *Φ*_F_ relative to *tert*-butyl analogues, suggesting enhanced non-radiative decay *via* increased π-conjugation and possible charge transfer involving sulfur 3p orbitals. This interpretation agrees with the findings of Kobayashi *et al.*^55^ who demonstrated that thioalkyl substitution slightly decreases the fluorescence efficiency by extending π-delocalization through 3p-π orbital mixing. These results confirm that both the central metal ion and the substituted group effect cooperatively regulate the excited states of the Pc macrocycle.

### 3.3. Biological studies

Stock solutions of *tert*-butyl-substituted Pcs (*t*BuH_2_Pc, *t*BuZnPc, *t*BuNiPc, and *t*BuCuPc) and their thio-substituted derivatives (S-*t*BuH_2_Pc, S-*t*BuZnPc, S-*t*BuNiPc, and S-*t*BuCuPc) were prepared in DMSO. Complete dissolution was ensured by performing ultrasonic treatment three times for 10 min each. The dissolved stock solutions were then diluted with pure water to obtain the desired concentrations for biological studies.

#### 3.3.1 Effect of *tert*-butyl and S-*tert*-butyl group-substituted pcs on α-amylase activity

**Fig. 4:**
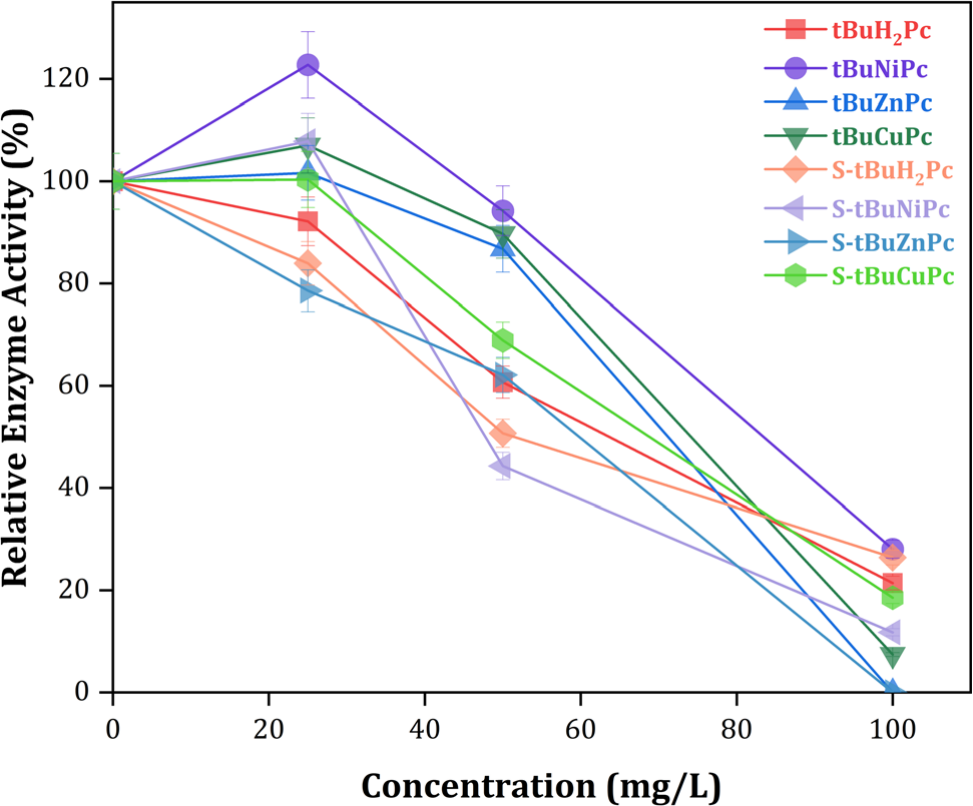
Effect of the Pc derivatives functionalized with *tert*-butyl and S-*tert*-butyl groups on the α-amylase activity.

Diabetes is a chronic condition where the body struggles to regulate blood sugar due to either excessive insulin production or the inability of insulin to effectively control glucose levels. Insulin, a hormone produced by the pancreas's beta cells, plays a crucial role in regulating, absorbing, and utilizing blood glucose.^56,57^ Patients with diabetes develop a condition called “insulin resistance”; here, body cells become resistant to insulin and the effect of insulin decreases. This leads to a buildup of blood sugar and type 2 diabetes.^58^ The causes of diabetes are various; its management varies depending on the type of disease and needs to be investigated in detail. DM is also a universal health crisis, and different agents are used in its treatment, but these drugs are high-priced with potentially severe side effects.^59^ α-Amylase is a key enzyme responsible for the hydrolysis of dietary starch into glucose. Since high enzyme activity leads to rapid glucose absorption and hyperglycemia, the inhibition of this enzyme is a desirable mechanism for antidiabetic agents to control postprandial blood glucose levels. The effects of the Pcs derivatives functionalized with *tert*-butyl and S-*tert*-butyl groups on α-amylase activity were also investigated, and the results are presented in Fig. 4.

The amylolytic effects of the Pc derivatives were highly dependent on the central metal ion and concentration. Throughout the concentration range, *t*BuNiPc demonstrated the highest α-amylase activation compared to both the S-*t*But and *t*But Pc derivatives. Within the *t*But series, metal complexes generally yielded higher activity than *t*BuH_2_Pc. This trend remained consistent for the S-*t*BuPc series, except for S-*t*BuZnPc at 20 mg L^−1^ and S-*t*BuNiPc at 50 mg L^−1^. On the whole, the *t*BuPc derivatives enhanced the α-amylase activity more effectively than the corresponding S-*t*BuPc derivatives. The pronounced activation caused by *t*BuNiPc at low concentrations highlights its prospects as an activator in starch processing. Conversely, at higher concentrations (50–100 mg L^−1^), significant inhibition of α-amylase was observed for most compounds, suggesting their potential development as antidiabetic agents.

These dose-dependent effects may be attributed to different mechanisms. At low doses, some compounds may facilitate enzyme–substrate interactions by acting as a bridge, thereby enhancing the enzyme activity. At higher concentrations, inhibition may occur through binding to the active site or by inducing conformational changes in the enzyme structure. These findings are consistent with previous reports.^35,60^ Özçeşmeci *et al.* (2024) investigated the α-amylase inhibitory potential of carbon, carbon–boron quantum dots and Cu(ii)Pc derivative and reported that inhibition increased with the increasing concentration.^35^ Similarly, Günsel *et al.* (2022) demonstrated that Pc derivatives can effectively inhibit both α-amylase and α-glucosidase activities, suggesting their potential as oral antidiabetic agents.^61^

Overall, the findings of the present study indicate that the synthesized Pc derivatives, particularly *t*BuNiPc, exert a dual modulatory effect on α-amylase activity, enhancing enzymatic action at low concentrations while inducing significant inhibition at elevated concentrations. Consequently, these compounds represent promising candidates for further evaluation, offering potential utility in industrial biotechnology at lower concentrations and as therapeutic agents in biomedical applications at higher doses.

#### 3.3.2. DNA cleavage results

DNA plays an important role as a biological target in drug development; therefore, the DNA cleavage activities of the Pc derivatives were examined. The results are given in Fig. S24. It is observed that the interaction between the compounds and supercoiled plasmid DNA varies with changes in the compound concentrations. Complete DNA fragmentation was observed at all concentrations of compounds *t*BuNiPc, S-*t*BuH_2_Pc, S-*t*BuNiPc, S-*t*BuZnPc, and S-*t*BuCuPc, whereas compound *t*BuH_2_Pc (Fig. S25a; Lane 2 and 3) caused double-strand breaks at 50 and 100 mg L^−1^ and complete fragmentation at 200 mg L^−1^. Additionally, *t*BuZnPc (Fig. S25b; Lane 2) and *t*BuCuPc (Fig. S25b; Lane 5) induced single-strand breaks at 50 mg L^−1^, while complete DNA fragmentation was observed at 100 and 200 mg L^−1^.

Celep *et al.* informed that they tested the DNA cleavage activities of zinc and indium phthalocyanines, and it was reported that both ZnPc and InPc induced double-strand breaks at 50 mg L^−1^ and caused complete DNA fragmentation at 200 mg L^−^.^62^ Harmandar *et al.* investigated the DNA cleavage activities of zinc and magnesium Pcs substituted with tetrahydropyrimidone groups.^60^ Their findings indicated partial strand breakage at 25, 50, and 100 mg L^−1^, with complete DNA degradation observed only at higher doses. Similarly, Demirbaş *et al.* reported DNA cleavage activity for triazole-substituted zinc phthalocyanines; however, complete fragmentation occurred only at relatively high concentrations (≥100 mg L^−1^).^63^

Our study showed that all the Pc derivatives caused complete DNA fragmentation at all tested doses (100–200 mg L^−1^). Notably, compound *t*BuH_2_Pc led to double-strand breaks at 50 and 100 mg L^−1^, and full fragmentation at 200 mg L^−1^. DNA cleavage activity can be explained by the presence of aromatic rings that can stack DNA bases and oxygen and nitrogen atoms that can form hydrogen bonds with DNA.^64^ This consistent and concentration-independent cleavage behavior highlights the superior DNA-damaging efficiency of our Pc derivatives compared to previously reported analogues, suggesting their strong potential as versatile candidates in the design of next-generation therapeutic agents.

#### 3.3.3. Antimicrobial activity results

The antimicrobial activities of the Pc derivatives were evaluated using the microdilution method, and the minimum inhibitory concentration (MIC) values are presented in Table 2. All tested compounds exhibited notable antimicrobial activity against various microorganisms. In general, the S-*t*BuPc derivatives showed higher antimicrobial activity than their *t*BuPc analogues, particularly against *S. aureus* and *P. aeruginosa*. Moreover, the thio-substituted Pc derivatives demonstrated improved effectiveness against Gram-negative bacteria, suggesting that peripheral donor-type substituents may enhance the biological performance of Pc derivatives. As shown in Table 2, *L. pneumophila* subsp. *pneumophila* was the most sensitive microorganism among those tested.

**Table 2:** Antimicrobial activity

Microorganism	MIC values (mg L^−1^)									
	*t*BuH_2_Pc	*t*BuNiPc	*t*BuZnPc	*t*BuCuPc	S-*t*BuH_2_Pc	S-*t*BuNiPc	S-*t*BuZnPc	S-*t*BuCuPc	aFlc	bAmp
*E. coli*	32	32	16	32	32	8	16	16	—	0.5
*P. aeruginosa*	64	64	32	64	64	16	8	16	—	0.5
*L. pneumophila*	64	16	32	64	8	4	4	4	—	0.5
*E. hirae*	64	32	32	32	16	16	16	16	—	0.5
*E. faecalis*	128	64	64	64	64	16	32	32	—	0.5
*S. aureus*	128	64	64	64	32	32	32	64	—	1
*C. parapsilosis*	128	64	64	128	128	64	32	64	1	—
*C. tropicalis*	128	64	128	128	128	64	64	128	1	—

a Flc: Flucozanole and.

b Amp: Ampicillin.

The antimicrobial properties of the Pc compounds were found to be comparable with those reported for other Pc derivatives in the literature.^35,65–68^ Previous studies have also highlighted the antimicrobial potential of Pc derivatives. For instance, the *in vitro* aPDT activity of ZnPc derivatives and their nanoconjugates (3@N,S-GQDs and 4@N,S-GQDs) against the Gram-positive bacterium *S. aureus* demonstrated that sulfur-containing Pc derivatives exhibited enhanced antibacterial activity, consistent with the findings of the present study.^65^ In another study, the antimicrobial activities of carbon quantum dots (CQD), carbon-boron quantum dots (CBQD), CuPc-G, CQD/CuPc-G, and CBQD/CuPc-G were investigated, and several compounds showed strong antibacterial activity against both Gram-positive (*E. hirae* and *E. faecalis*) and Gram-negative (*E. coli*, *P. aeruginosa*, and *B. subtilis*) bacteria.^35^ Similarly, studies on Pc derivatives under dark and light conditions revealed that *S. aureus* was more sensitive under dark conditions compared with *E. coli* and *P. aeruginosa*. Gerasymchuk reported that zirconium Pc composites did not significantly affect the bacterial viability.^66^ In another study, Türkan *et al.* evaluated the antimicrobial properties of Co(ii) and Zn(ii) Pc derivatives against *E. coli* and *S. aureus*, and they reported that Zn(ii)Pc showed high activity against *E. coli*, while Co(ii)Pc derivatives exhibited moderate activity against *E. coli* and inhibited *S. aureus* growth only at 125 µM.^68^

Based on these results, the tested compounds particularly S-*t*BuH_2_Pc, S-*t*BuZnPc, S-*t*BuNiPc, and S-*t*BuCuPc may represent promising candidates for further antibacterial drug research and could serve as potential starting points for the development of new antimicrobial agents after more detailed investigations.

#### 3.3.4. Antibiofilm and aPDT results

Biofilms are communities where microbial cells form complex and dynamic structures organized in a matrix produced by extracellular polymers and other biomolecules. These structures include different species and genotypes and evolve by interacting with their environment.^65^ Biofilms are a common form of microbial life on earth and drive the functioning of ecosystems.^66^ They also impact global issues such as climate change, health issues, and antimicrobial resistance. Therefore, understanding and controlling the activities of biofilms may provide scientific and societal benefits.^67^ In our study, the antibiofilm abilities of the Pcs were examined against *S. aureus* and *P. aeruginosa*, microorganisms known to form biofilms, using the crystal violet binding assay. All the obtained results of antibiofilm activity with and without PDT are indicated in Fig. S21–S24. The biofilm inhibition results indicated a clear concentration-dependent effect. The antibiofilm activities of the Pc derivatives against *S. aureus* ranged from 48.79% to 69.47% at 10 mg L^−1^ and increased markedly to 79.63–100% at 15 mg L^−1^. Similarly, strong antibiofilm activity was observed against *P. aeruginosa*, with inhibition values reaching up to 100% at 15 mg L^−1^. The comparison of the results for both microorganisms revealed that S-*t*BuH_2_Pc, S-*t*BuZnPc, S-*t*BuNiPc, and S-*t*BuCuPc exhibited superior antibiofilm performance compared with the other derivatives.

These findings are consistent with previous studies.^4,35,69^ For example, the antibiofilm activities of CQD, CBQD, CuPc-G, CQD/CuPc-G, and CBQD/CuPc-G at 50 mg L^−1^ were reported to be 75.7–95.1% for *S. aureus*, while lower activities were observed against *P. aeruginosa* (29.99–57.47%).^35^ Farajzadeh *et al.* (2022) reported the antibiofilm activities of various metal Pcs (Pc, ZnPc, CuPc, CoPc, InPc, and LuPc) against *S. aureus* at 62.5 mg L^−1^ as 55.18%, 76.78%, 64.59%, 72.27%, 65.33%, and 68.8%, respectively. The antibiofilm activities of these Pcs against *P. aeruginosa* were found to be 29.99%, 57.47%, 49.31%, 45.92%, 48.56%, and 43.71%, respectively.^4^ Openda and Nyokong also reported that quaternized Pc derivatives exhibited enhanced antibiofilm activity against *E. coli* and *S. aureus*.^69^ Compared with previous reports, the Pc derivatives investigated in this study demonstrated strong biofilm inhibition against the tested microorganisms. These findings suggest that the tested Pc compounds may provide valuable insights for future studies and could serve as promising candidates for the development of biofilm-preventive agents after further detailed investigations and *in vivo* studies.

**Fig. 5:**
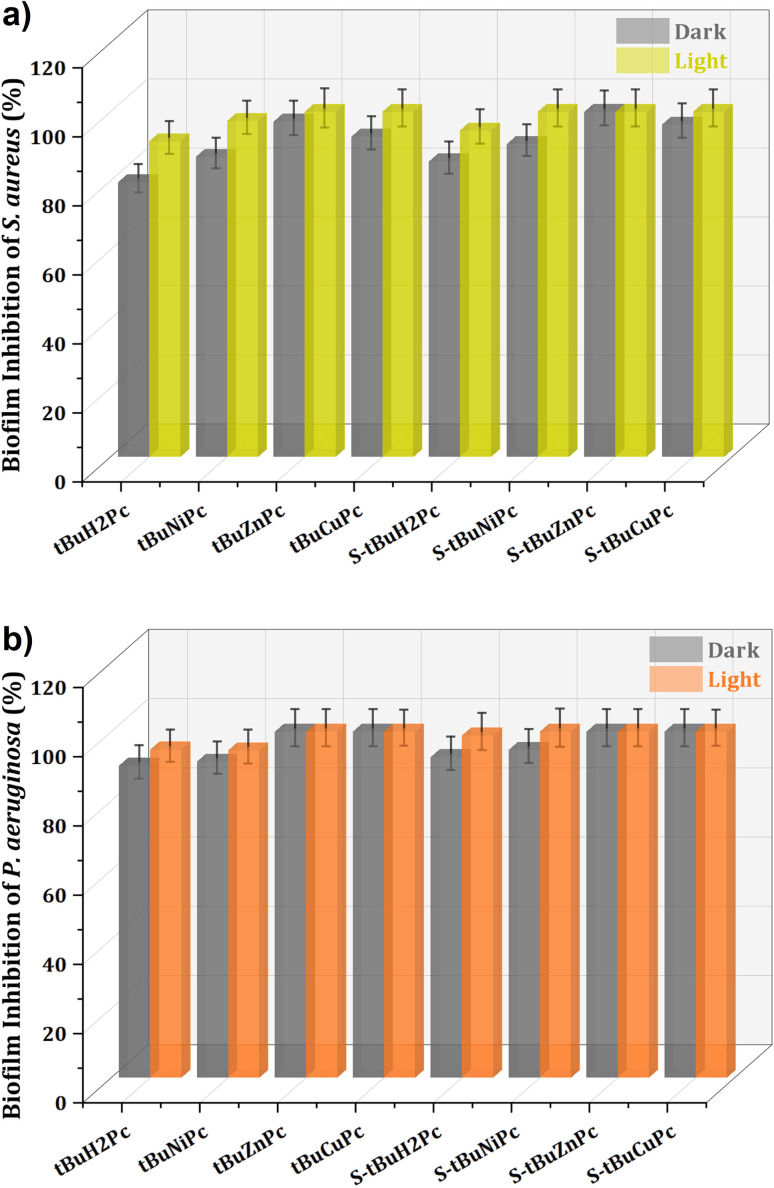
Biofilm inhibition activity of Pcs against (a) *S. aureus* and (b) *P. aeruginosa* at 15 mg mL^−1^ concentration under dark and LED light conditions.

Although PDT has been established in microbiology for over a century, its application has been more prevalent in oncology and ophthalmology. In 1975, the initial clinical report of aPDT for treating lesions caused by the Herpes simplex virus was documented.^70^ However, the treatment was later criticized for being ineffective and causing side effects.^71^ In recent years, as bacteria have gained resistance to antibiotics, aPDT has garnered renewed interest as a promising alternative, particularly for multidrug-resistant infections. aPDT has been shown to effectively eradicate resistant microbes in clinical settings. While mechanisms by which bacteria could develop resistance to PDT have been proposed, no such resistance has been observed to date.^72^ It is therefore seen as an alternative method to prevent biofilm formation. In this study, the antibiofilm activities of Pcs with PDT were examined against *S. aureus* and *P. aeruginosa*, which are known to form biofilms, and the results are shown in Fig S20–24. The antibiofilm activities of Pc derivatives under PDT conditions were evaluated against *S. aureus* and *P. aeruginosa*. For *S. aureus*, the antibiofilm activities were 58.69%, 75.42%, 94.71%, 83.66%, 62.37%, 80.61%, 98.55%, and 90.36% at 10 mg L^−1^, respectively. At 15 mg L^−1^, the inhibition values increased markedly, reaching 91.48%, 97.33%, and up to 100% for several derivatives. Similarly, strong antibiofilm activity was observed against *P. aeruginosa*, where inhibition values reached 94.57–100% at 15 mg L^−1^. When the results for both microorganisms were compared, S-*t*BuH_2_Pc, S-*t*BuZnPc, S-*t*BuNiPc, and S-*t*BuCuPc exhibited the highest antibiofilm efficacy (Fig. 5). These findings are consistent with previous reports on the antibiofilm potential of Pc derivatives. Farajzadeh *et al.* investigated metallophthalocyanine compounds and reported antibiofilm activities of these compounds as 55.18% at 62.5 mg L^−1^ and 75.31% at 250 mg L^−1^.^4^ When PDT was applied under similar conditions, the inhibition increased significantly, reaching 90.67% at 62.5 mg L^−1^ and 98.74% at 250 mg L^−1^. Similarly, Carmello *et al.* demonstrated that red LED-mediated aPDT using chloro-aluminum Pc derivatives effectively inhibited biofilm formation in *Candida albicans*, reducing adhesion and virulence during the *in vivo* treatment of oral candidiasis.^73^ These studies highlight the significant enhancement of antibiofilm activity when PDT is applied.

The mechanism of aPDT involves three key components: a photosensitizer (PS), light of an appropriate wavelength, and molecular oxygen. Upon irradiation of PS, the ground-state photosensitizer is excited to a singlet excited state and subsequently converted to a longer-lived triplet state through intersystem crossing. In this excited triplet state, the PS can transfer energy to molecular oxygen, generating reactive oxygen species (ROS), particularly singlet oxygen (^1^O_2_). These highly reactive species cause oxidative damage to essential cellular components such as the bacterial cell wall, membrane, and DNA, ultimately leading to microbial cell death.^74^ Since biofilm-associated infections are difficult and costly to treat, the development of new antimicrobial agents and alternative therapeutic strategies has become increasingly important. Considering the growing threat of antimicrobial resistance, expanding research in this area is essential. The results of the present study suggest that the synthesized Pc derivatives exhibit strong antibiofilm potential and may serve as promising candidates for further investigation as photodynamic antibiofilm agents.

#### 3.3.5. Microbial cell viability inhibition

**Fig. 6:**
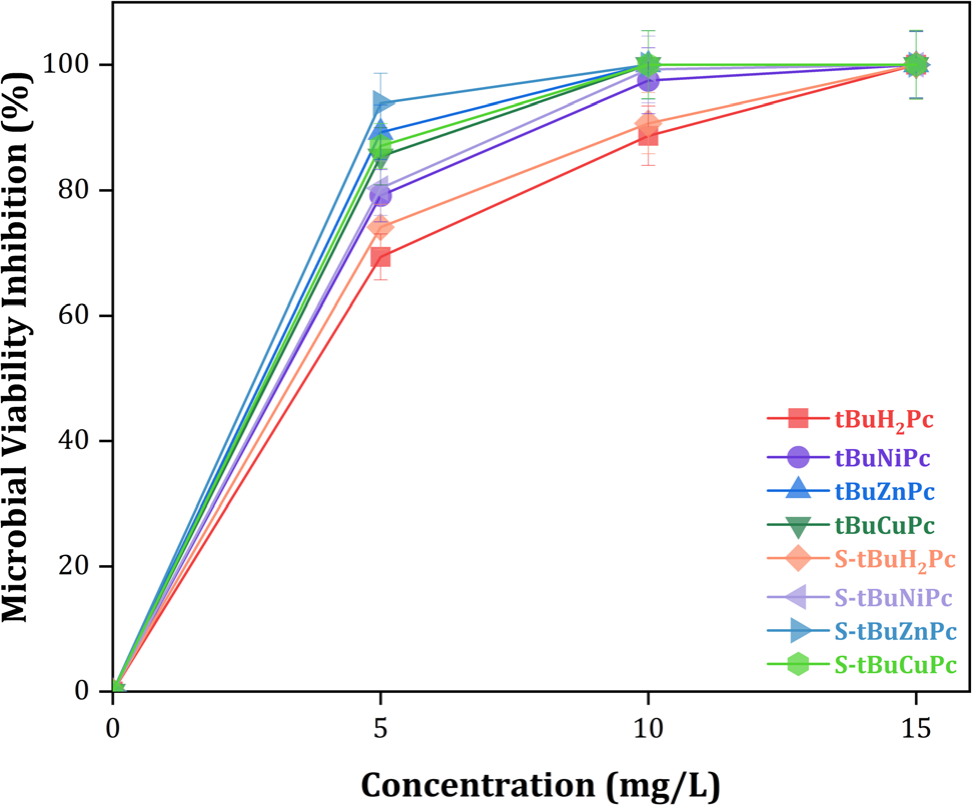
Microbial cell viability inhibition.

Hospital-acquired infections affect millions of people worldwide, often leading to unnecessary or prolonged treatment. The immune system naturally handles the infection in immunocompetent patients.^75^ However, this system faces different situations in patients with immunodeficiency diseases. For example, various pathogens can spread into the bodies of these patients and cause a wide variety of diseases. The pathogenic cell viability abilities of Pcs were examined against a Gram (−)ve bacteria of *E. coli* in this research. The results obtained at the end of the study are listed in Fig. 6. While *t*BuZnPc, *t*BuCuPc, S-*t*BuZnPc and S-*t*BuCuPc were observed to completely inhibit the growth of *E. coli* at a concentration of 10 mg L^−1^, all the samples also completely inhibited the growth of *E. coli* at a concentration of 15 mg L^−1^.

Mohamed *et al.* reported that the Cu(ii) Pc derivative showed excellent inhibitory activities against *B. cereus* and *P. aeruginosa* when the inhibitory activity was tested against multi-drug-resistant *P. aeruginosa* and *B. cereus*.^75^ Hamouda *et al.* prepared a graphene-ZnO hybrid nanocomposite and its antibacterial properties were investigated, it was shown that bacterial growth was significantly inhibited.^76^ Considering the results obtained from our study, it can be said that all Pcs studied can be very strong candidates for pathogenic cell inhibitors.

### 3.4. Theoretical studies

#### 3.4.1. DFT calculation results

**Fig. 7:**
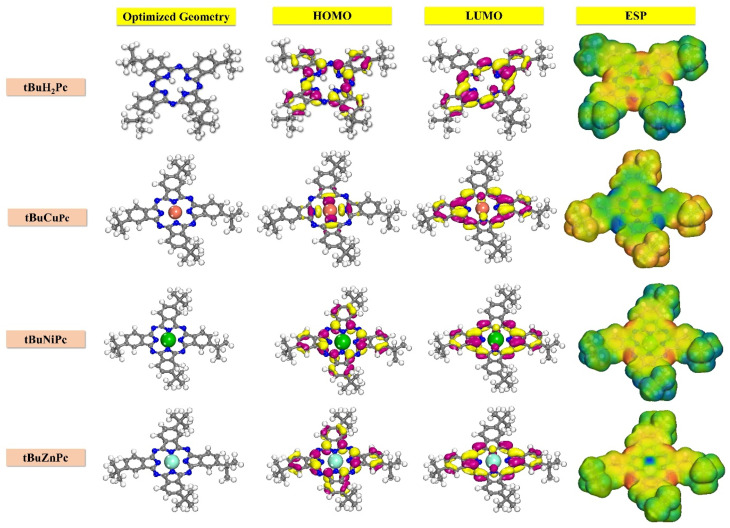
Optimized geometries, HOMOs, LUMOs, and electrostatic potential (ESP) maps of the *t*-butyl-substituted phthalocyanine derivatives. From top to bottom: metal-free *t*BuH_2_Pc, *t*BuCuPc, *t*BuNiPc and *t*BuZnPc complexes. The HOMO and LUMO plots reveal the frontier orbital distributions influenced by the central metal coordination, while the ESP maps depict the variations in electron density, highlighting the regions of electrophilic and nucleophilic reactivity.

**Fig. 8:**
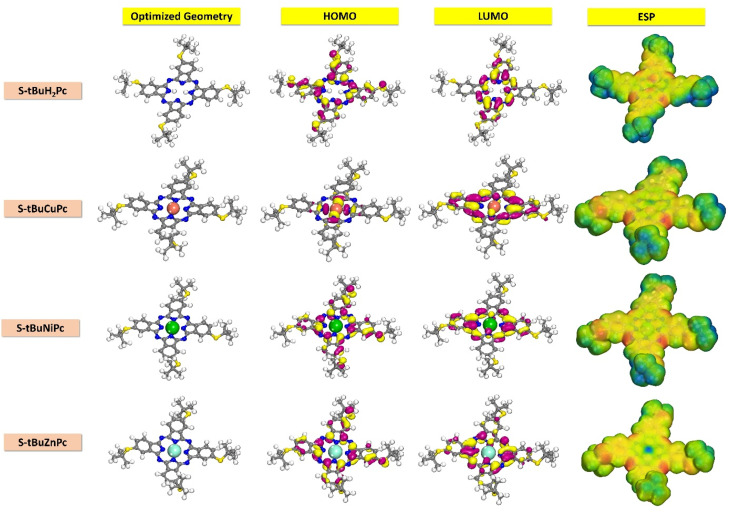
Optimized geometries, HOMOs, LUMOs, and electrostatic potential (ESP) maps of the *t*-butyl-substituted phthalocyanine derivatives. From top to bottom: metal-free S-*t*ButH_2_Pc, S-*t*BuCuPc, S-*t*BuNiPc and S-*t*BuZnPc complexes. The HOMO and LUMO plots reveal the frontier orbital distributions influenced by the central metal coordination, while the ESP maps depict the variations in electron density, highlighting the regions of electrophilic and nucleophilic reactivity.

The parameters used for reactivity analysis of chemical species within the scope of Conceptual Density Functional Theory are used to explain the biological activities of molecular systems, and in light of these parameters, drug activities of chemical systems can be predicted with the support of Molecular Docking analyses. Molecular orbital energies, *E*_HOMO_ and *E*_LUMO_, provide great facilities to computational chemists for the estimation of the electron-donating and electron-accepting capabilities of the molecules. Table S1 in the SI presents the calculated reactivity descriptors of the studied chemical systems. Fig. 7 and 8 visually reflect the optimized structure, HOMO, LUMO, and ESP images for the same chemical systems. It is known that the molecules with high HOMO energy are the chemical systems that can easily donate electrons and act as good Lewis bases. However, molecules with low LUMO energy are strong Lewis acids with high electron affinity. Electronegativity concept introduced by Pauling^77^ indicates the tendency of chemical systems to attract electrons. When the values given in the SI (Table S1) are examined, it is seen that the compounds with the lowest electronegativity are *t*BuCuPc and S-*t*BuCuPc, while the most electronegative compound is S-*t*BuZnPc. Chemical hardness,^78,79^ one of the most important parameters reflecting the stability of chemical systems, represents the resistance to change in the electron distribution of an atom, ion, or molecule. The relation between this concept and stability has been reported in the light of the Maximum Hardness Principle.^80^ This principle states that the molecules with lower hardness are softer and more reactive. In other words, as hardness increases, stability will increase. Therefore, among the studied chemical systems, the ones with the highest hardness or stability are *t*BuNiPc and *t*BuZnPc, while the most reactive and soft ones are *t*BuCuPc and S-*t*BuCuPc. When looking at the chemical hardness values, one of the striking situations in the table is that when two different complexes of the same metal are considered, the complex containing sulfur is softer and more reactive than the other. This situation is also compatible with the Hard and Soft Acid-Base Principle.^81^ Because, according to the hard and soft acid-base approach, the S^2−^ ion and many molecules containing S in their structure are among the soft bases. Another electronic structure principle considered in chemical reactivity analysis studies is the Minimum Electrophilicity Principle.^82^ According to this electronic structure principle, highlighting the relation with electrophilicity indexes, *ω*_1_ and *ω*_2_, electrophilicity is a chemical property minimized in stable states. According to the calculated electrophilicity values, as in the Maximum Hardness Principle, the Minimum Electrophilicity Principle predicts that the *t*BuCuPc and S-*t*BuCuPc complexes are more reactive than the other complexes, while the *t*BuNiPc and *t*BuZnPc complexes are the most stable of the studied complexes. In the experimental part of the study, the biological activity of the synthesized compounds was examined in many aspects. In the part where molecular docking analyses were performed, the interactions of the compounds with the alpha-amylase enzyme were analyzed, and their amylolytic activities were theoretically predicted. Both experimental studies and molecular docking analyses have shown that thio-substitution with lower chemical hardness values and higher electrophilicity index values interacts more strongly with the alpha-amylase enzyme and exhibits higher amylolytic activity.

Among the *tert*-butyl-substituted derivatives, *t*BuCuPc displayed a noticeably different frontier orbital and ESP distribution compared with the other complexes. This behavior can be associated with the open-shell d^9^ Cu(ii) center, which perturbs the electronic distribution of the Pc macrocycle more strongly than Zn(ii), Ni(ii), or the metal-free core. In contrast, the S-*t*BuCuPc derivative showed a distribution more consistent with the remaining S-*t*Bu-substituted complexes, suggesting that the sulfur-containing peripheral groups promote electron-density delocalization and reduce the apparent Cu-centered electronic asymmetry.

The experiments conducted showed that S-*t*ButH_2_Pc, S-*t*BuCuPc, S-*t*BuNiPc, and S-*t*Bu-ZnPc are more effective than the other ingredients in terms of antimicrobial activity. When the DNA cleavage properties of the studied compounds were evaluated, it was reported that, in addition to the four compounds mentioned as effective in terms of antimicrobial activity, *t*BuCuPc showed full cleavage activity at all concentrations of DNA. These observations prove that adding S to the structure increases the interaction with biological systems, and the biological activity of soft chemical systems is higher.

#### 3.4.2. Molecular docking results

**Fig. 9:**
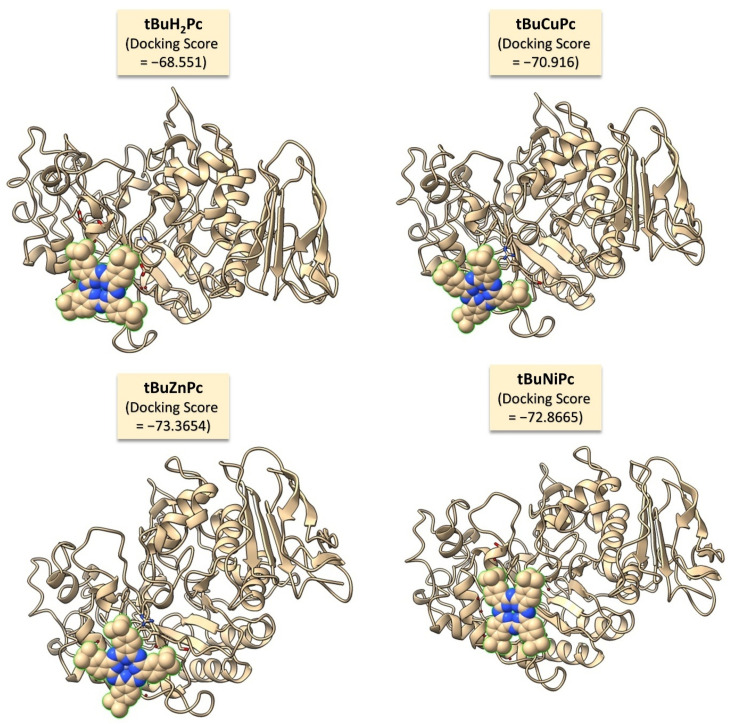
Binding conformations of the *t*-butyl-substituted phthalocyanine derivatives docked into the active site of the human pancreatic α-amylase (PDB ID: 1HNY). The ligands, *t*BuH_2_Pc, *t*BuCuPc, *t*BuZnPc, and *t*BuNiPc, are shown in space-filling representations. Docking scores for each complex are indicated, reflecting their predicted binding affinities. All structures were visualized using ChimeraX.^83^

Molecular docking simulations were performed to compare the binding behavior of the *tert*-butyl- and *tert*-butylthio-substituted phthalocyanines toward human pancreatic α-amylase. As shown in Fig. 9, all investigated Pc derivatives adopted broadly similar binding orientations and were mainly positioned at the enzyme surface/active-site entrance rather than deeply inserted into the catalytic pocket. This behavior is consistent with the large, planar, and hydrophobic character of the Pc macrocycle. Therefore, the docking results should be interpreted primarily in terms of comparative binding tendency rather than as evidence for highly specific residue-level inhibition. The calculated MolDock scores indicated favorable binding for all compounds, with moderate differences depending on the central metal ion and peripheral substituent. In general, thio-substituted derivatives showed slightly improved binding affinities compared with their *tert*-butyl analogues, which may be associated with the higher polarizability of the sulfur-containing substituents and their ability to promote additional hydrophobic and van der Waals contacts with the protein surface. Among the derivatives, the Cu(ii), Zn(ii), and Ni(ii) complexes displayed comparable binding modes, while the metal-free compounds showed similar surface association but slightly different interaction patterns. Overall, the docking results support the experimental α-amylase activity data by suggesting that these Pc derivatives can interact favorably with α-amylase, although their large macrocyclic structures lead to similar binding poses.

##### 3.4.2.1. Comparative molecular docking analysis of *t*-butyl-Phthalocyanine derivatives

To gain insights into the inhibitory mechanisms of synthesized *t*-butyl-substituted phthalocyanines, we performed molecular docking simulations of the Cu(ii), Zn(ii), Ni(ii), and metal-free derivatives against human pancreatic α-amylase (PDB ID: 1HNY). All compounds demonstrated favorable binding affinities and engaged in multifaceted interactions with key residues in the enzyme's active site. The docking scores, docking poses, and interaction profiles are discussed in detail in Fig. 9.

###### 3.4.2.1.1. *t*BuCuPc

The copper-centered phthalocyanine exhibited a MolDock score of −70.916, indicative of strong binding affinity and favorable energetics. The total interaction energy was calculated as −73.815 kcal mol^−1^, primarily arising from steric interactions (−41.438 kcal mol^−1^) within the binding pocket. While no classical hydrogen bonding was detected, the ligand formed significant water-mediated interactions (−29.052 kcal mol^−1^), involving structured waters such as HOH643 and HOH599. These bridge contacts contributed to the stability and orientation of the ligand. Residue-level analysis identified Tyr2 (−13.98 kcal mol^−1^) as a key stabilizer, likely due to π–π stacking with the macrocyclic core. Additional supportive contacts were observed with Gly249, Phe248, Arg291, and Lys208, forming a favorable interaction network around the flat aromatic surface of the phthalocyanine. The ligand's internal energy strain was minimal (−3.325 kcal mol^−1^), and torsional strain remained negligible (0.289 kcal mol^−1^), indicating that the ligand retained structural integrity upon binding. These findings correlate well with the compound's potent α-amylase inhibition observed experimentally.

###### 3.4.2.1.2. *t*BuZnPc

The Zn(ii)-containing derivative demonstrated the strongest overall binding, with a MolDock score of −75.365 and a re-rank score of −43.439. The interaction energy profile revealed dominant steric contributions (−41.455 kcal mol^−1^), alongside extensive water–ligand interactions (−29.363 kcal mol^−1^). Key residues such as Tyr2, Phe248, and Gly249 were heavily involved, supporting strong π–π stacking and hydrophobic interactions.

Tyr2 again emerged as the primary anchor (−13.79 kcal mol^−1^), emphasizing its recurrent role across all active phthalocyanine derivatives. The internal strain was modest (−4.547 kcal mol^−1^), and torsional strain was low (0.209 kcal mol^−1^), indicating structural adaptability. Although direct coordination with Zn(ii) was not observed, the metal center likely contributed to macrocyclic rigidity and enhanced π-delocalization. These computational results align closely with the experimental data, where *t*-but-PcZn showed excellent α-amylase inhibition.

###### 3.4.2.1.3. *t*BuNiPc

The Ni(ii) derivative exhibited a strong MolDock score of −72.8665, confirming a high binding affinity. The protein–ligand interaction energy reached −54.521 kcal mol^−1^, primarily from steric (−52.898 kcal mol^−1^) and π–π stacking forces. Residues Tyr2 (−18.22 kcal mol^−1^) and Phe248 (−10.90 kcal mol^−1^) again played dominant roles in ligand anchoring. Notably, four hydrogen bonds were detected in this complex, including two water-mediated bridges, providing moderate polar stabilization (−1.624 kcal mol^−1^ total). Structured waters like HOH700 and HOH618 further contributed through bridging contacts, resulting in −17.991 kcal mol^−1^ of water–ligand interaction energy. The ligand displayed excellent conformational stability (internal strain: −1.158 kcal mol; torsional strain: 0.065 kcal mol^−1^), and the central Ni(ii) ion contributed −4.50 kcal mol^−1^ to macrocyclic rigidity, although no direct coordination with the protein was noted.

###### 3.4.2.1.4. *t*BuH_2_Pc

An alternative docking pose of the metal-free phthalocyanine derivative yielded a MolDock score of −68.5525, slightly lower than the Cu(ii)-centered analogue but still indicative of biologically meaningful binding. The protein–ligand interaction energy was −65.891 kcal mol^−1^, mainly driven by steric contacts (−63.387 kcal mol^−1^), with minor contributions from hydrogen bonding (−2.504 kcal mol^−1^). Two weak hydrogen bonds were formed with Asp246 and Arg291, contributing to binding stability. Water-mediated interactions also played a crucial role, with −16.526 kcal mol^−1^ attributed to bridging from HOH599, HOH637, and HOH700. These waters helped orient the ligand between key residues such as Gly249, Phe248, and Tyr2. Despite a slightly higher internal energy (13.865 kcal mol^−1^), the ligand showed minimal torsional distortion (0.285 kcal mol^−1^) and maintained favorable π-stacking with residues like Phe248 (−17.28 kcal mol^−1^) and Gly249 (−14.03 kcal mol^−1^). The lack of a central metal did not prevent effective binding, though the lower score suggests a somewhat reduced stabilization relative to its metallated counterparts.

##### 3.4.2.2 Insights into the comparative binding of Thio-substituted phthalocyanine derivatives

**Fig. 10:**
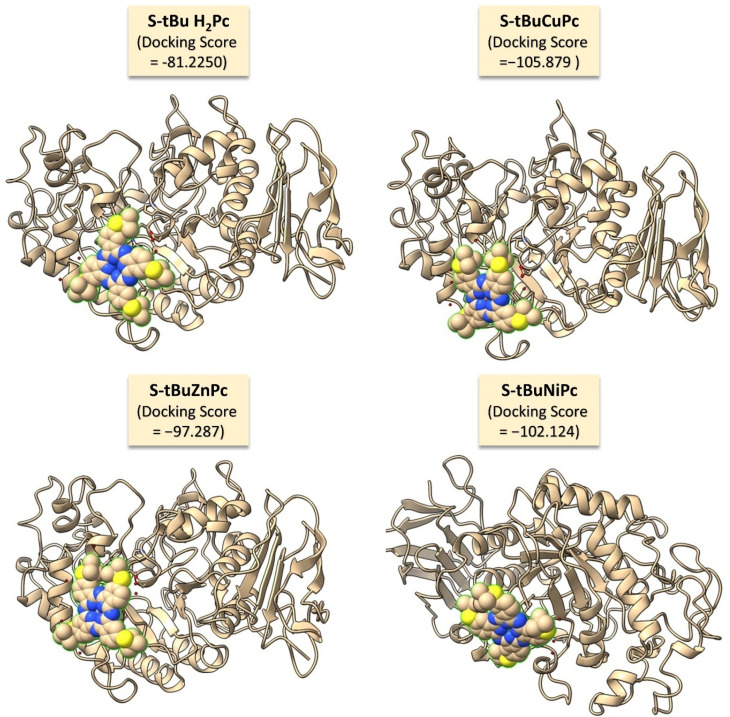
Binding conformations of the *t*-butyl-S-substituted phthalocyanine derivatives docked into the active site of the human pancreatic α-amylase (PDB ID: 1HNY). The ligands, S-*t*BuH_2_Pc, S-*t*BuCuPc, S-*t*BuZnPc, and S-*t*BuNiPc are shown in space-filling representations. Docking scores for each complex are indicated, reflecting their predicted binding affinities.

Among the thio-substituted phthalocyanine derivatives examined (Fig. 10), S-*t*BuH2Pc showed a promising docking profile, recording a MolDock score of −81.225. This indicates a stable and energetically favorable interaction with human pancreatic α-amylase (PDB: 1HNY). The ligand's binding was mainly driven by steric interactions (−61.194 kcal mol^−1^), reflecting its extended aromatic structure and bulky *tert*-butyl and thio-*tert*-butyl substituents. Hydrogen bonding contributed modestly (−2.941 kcal mol^−1^), involving five hydrogen bonds—two direct and three water-mediated—connecting with residues like Asp290, Ser245, and Arg291, which flank the catalytic groove.

Water molecules (*e.g.*, HOH599, HOH700, and HOH773) played a key role in mediating these interactions, especially with residues like Gly249 and Tyr2, facilitating a snug fit within the binding pocket. Despite some torsional strain (3.374 kcal mol^−1^) from side-chain flexibility, the macrocycle maintained alignment within the groove, forming strong π–π stacking with Tyr2 (−18.61 kcal mol^−1^) and Phe248 (−16.54 kcal mol^−1^). Other supportive contacts came from Arg291 and Gly249, reaffirming their roles in stabilizing aromatic ligands. The S-*t*BuZnPc outperformed its metal-free counterpart with a MolDock score of −97.287. The overall interaction energy (−61.979 kcal mol^−1^) was again dominated by steric contributions (−61.349 kcal mol^−1^), but also featured water-mediated stabilization (−19.550 kcal mol^−1^). Three structured water molecules—HOH599, HOH700, and HOH823—formed hydrogen bonds that enhanced the ligand's orientation and fit. Key residues involved remained consistent: Tyr2 (−16.45 kcal mol^−1^), Phe248 (−11.20 kcal mol^−1^), and Arg291 (−12.64 kcal mol^−1^). The Zn(ii) ion, while not directly coordinating to residues, likely contributed intramolecular stabilization (−2.29 kcal mol^−1^) of the macrocycle, supporting a favorable conformation. Notably, torsional strain was minimal (1.905 kcal mol^−1^), allowing side-chain flexibility without major distortion.

The copper-centered derivative (S-*t*BuCuPc) showed the highest binding affinity across all candidates, with a MolDock score of −105.879. The interaction energy (−64.989 kcal mol^−1^) stemmed primarily from steric effects (−64.543 kcal mol^−1^), with additional support from water-mediated interactions (−21.717 kcal mol^−1^). The ligand formed five hydrogen bonds, two of which were water-mediated through HOH599 and HOH700. Directional hydrogen geometries (*e.g.*, 176.8° D–H–A angle) suggested optimized polar stabilization. The same cluster of residues—Tyr2, Phe248, Gly249, Arg291, and Asp290—dominated interactions, with Cu(ii) playing a stabilizing role within the macrocyclic core (−2.29 kcal mol^−1^). The sulfur atoms likely enhanced shape complementarity and polarizability, aiding the deep and stable fit within the protein pocket. The Ni(ii)-centered S-*t*BuNiPc, closely trailing in performance (MolDock score of −102.124), exhibited a similar interaction profile. Its interaction energy (−64.989 kcal mol^−1^) and water bridging (−18.021 kcal mol^−1^) mirrored the Cu(ii) complex. The Ni(ii) ion also stabilized the macrocyclic structure (−2.01 kcal mol^−1^), and the same hotspot residues contributed to robust binding. Again, torsional strain was low (1.905 kcal mol^−1^), indicating that the ligand adapted well to the binding site.

## 4. Conclusion

In this study, Pcs were synthesized and characterized. In addition, some biological activities of newly synthesized Pcs, such as DNA fragmentation, antimicrobial, antibiofilm, antibiofilm activity with photodynamic therapy, antidiabetic, and cell viability functions, were investigated in the study. During the evaluation of biofilm inhibition activities of Pcs, *S. aureus* and *P. aeruginosa* were used as model microorganisms. Pcs effectively inhibited antibiofilm formation in both species, with inhibition increasing as the concentration of the compounds was increased. Furthermore, light irradiation enhanced the biofilm inhibition activity, showing even greater effectiveness post-irradiation. Regarding DNA cleavage, complete breakage was observed in the thio-substituted derivatives (S-*t*BuH_2_Pc, S-*t*BuZnPc, S-*t*BuNiPc, and S-*t*BuCuPc). All Pcs exhibited significant antimicrobial activity against the studied microorganisms, with samples S-*t*BuZnPc, S-*t*BuNiPc, and S-*t*BuCuPc showing particularly high activity. Additionally, all Pc compounds inhibited microbial cell viability against *E. coli* at every concentration tested, including very low concentrations. Based on these findings, it is believed that these compounds hold potential for diverse applications, particularly in medical research aimed at reducing microbial activity.

This study comparatively examined *tert*-butyl and thio-*tert*-butyl substituted phthalocyanines with different central metals and metal-free systems (H_2_, Zn^2+^, Cu^2+^, and Ni^2+^) to clarify how the sulfur donor atom and metal center influence their photophysical and biological properties. The incorporation of a sulfur atom into the phthalocyanine ring induced a 6–7 nm red shift in the Q-band, confirming its p-donor character and extended π-conjugation.

This modification slightly lowered the fluorescence quantum yields (*Φ*_F_) through the internal heavy-atom effect, favoring triplet-state formation, a key factor in photodynamic activity. Only the metal-free and Zn(ii) complexes were emissive, while Cu(ii) and Ni(ii) derivatives were completely quenched due to efficient intersystem crossing (ISC).

In all biological evaluations, including antibacterial, antibiofilm, DNA cleavage, and α-amylase inhibition assays, the S-*t*BuCuPc complex exhibited the highest activity, outperforming both its *tert*-butyl analogue and other metallated derivatives. This superior behavior arises from the synergistic effect of the sulfur atom and the paramagnetic Cu(ii) center, which enhances ROS generation and redox-mediated reactivity under light irradiation. In the theoretical section of the article, DFT-based calculations showed that copper complexes are more reactive than the other chemical systems studied. Chemical reactivity comparisons were done through the popular electronic structure principles of Conceptual DFT. Molecular Docking calculations made to check the power of the interactions of the compounds with the alpha-amylase enzyme proved that sulfur-containing systems interact more strongly with the enzyme in question. These systems, with lower chemical hardness and higher electrophilicity index values, also have higher amylolytic activity.

Overall, peripheral substitution of the S atom proved to be an effective design strategy for tuning excited-state dynamics and biological efficiency. The S-*t*BuCuPc derivative stands out as the most promising candidate for photodynamic, antimicrobial, and DNA-cleavage applications, combining efficient triplet-state formation with strong light-induced biological reactivity. Although the test compounds have shown effective biological activity, they cannot be used directly in the pharmaceutical industry. For this reason, further *in vitro* and *in vivo* studies are necessary to fully establish their safety profile for biomedical applications.

## Conflicts of interest

There are no conflicts to declare.

## Supplementary Material

RA-016-D6RA02238C-s001

## Data Availability

The data supporting this article have been included as part of the supplementary information (SI). Supplementary information: structure determination, additional structure diagrams, and copies of the NMR, FTIR, mass, and UV-vis spectra. See DOI: https://doi.org/10.1039/d6ra02238c.
