# Supplementary material for: A series of tert-butyl- and tert-butylthio-substituted phthalocyanine derivatives: biological activities, DFT calculations and molecular docking studies
Source: RSC Adv. 2026 Jul 13. Online ahead of print. doi: 10.1039/d6ra02238c (PMC13358459; doi:10.1039/d6ra02238c)
Supplement: RA-OLF-D6RA02238C-s001 [file RA-OLF-D6RA02238C-s001.pdf]

Supplementary Information (SI) for RSC Advances.
This journal is © The Royal Society of Chemistry 2026

Supporting Information

A series of tert-butyl and tert-butylthio -substituted phthalocyanine derivatives: biological activities, DFT calculations and molecular docking studies

Gülşah TOLLU[1], Gizem GÜMÜŞGÖZ ÇELİK[2], Gülenay TUNÇ[2], Sadin ÖZDEMIR[3], Savaş KAYA[4], Avni BERISHA[5], Ayşe Gül GÜREK[2], Devrim ATİLLA[2*]

[1]*Laboratory and Veterinary Health, Technical Science Vocational School, Mersin University, TR-33343 Mersin, Turkey*

[2]*Department of Chemistry, Gebze Technical University, 41400, Gebze, Kocaeli, Turkey*

[3]*Food Processing Programme, Technical Science Vocational School, Mersin University,33343 Yenisehir, Mersin, Turkey*

[4]*Sivas Cumhuriyet University, Faculty of Science, Department of Chemistry, 58140, Sivas/Turkey*

[5]*Department of Chemistry, Faculty of Natural and Mathematics Science, University of Prishtina, 10000 Prishtina, Kosovo*

1. Synthesis and Characterization of MPcs Derivatives 3

General procedure for the synthesis of 2(3),9(10),16(17),23(24)-Tetrakis(tert-butyl)metallo-phthalocyanines (tBuZnPc, tBuNiPc, tBuCuPc): 3

Synthesis of 2(3),9(10),16(17),23(24)-Tetrakis(tert-butyl)phthalocyanine (tBuH$_2$Pc): 4

General procedure for the synthesis of 2(3),9(10),16(17),23(24)-Tetrakis(tert-butylthio)metallo-phthalocyanines (S-tBuZnPc, S-tBuNiPc, S-tBuCuPc): 4

Fig. S1 FT-IR spectra of all tBuMPc derivatives. 6

Fig. S2 MALDI-TOF MS spectra of all tBuMPc derivatives obtained using the **DIT** matrix, with the $[M]^{+\cdot}$ molecular ion peaks marked on the spectra. 6

Fig. S3 ^{1}H NMR spectra for tBuNiPc in $CDCl_3$. 7

Fig. S4 ^{1}H NMR spectra for tBuZnPc in DMSO-*d6* 7

Fig. S5 ^{1}H NMR spectra for tBuH$_2$Pc in $CDCl_3$ 8

Fig. S6 FT-IR spectra of all S-tBuMPc derivatives.. 9

Fig. S7 MALDI-TOF MS spectra of all S-tBuMPc derivatives obtained using the **DIT** matrix, with the $[M]^{+\cdot}$ molecular ion peaks marked on the spectra. 10

Fig. S8 ^{1}H NMR spectra for S-tBuNiPc in $CDCl_3$. 11

Fig. S9 ^{1}H NMR spectra for S-tBuZnPc in $CDCl_3$... 11

Fig. S10 ^{1}H NMR spectra for $tBuH_2Pc$ in $CDCl_3$.. 12

Fig. S11 UV-Vis absorption spectra of **tBuCuPc** in THF solutions of 2–12 μM concentration range at room temperature. 12

Fig. S12 UV-Vis absorption spectra of **tBuZnPc** in THF solutions of 2–12 μM concentration range at room temperature. 13

Fig. S13 UV-Vis absorption spectra of **tBuNiPc** in THF solutions of 2–12 μM concentration range at room temperature. 13

Fig. S14 UV-Vis absorption spectra of **$tBuH_2Pc$** in THF solutions of 2–12 μM concentration range at room temperature. 14

Fig. S15 UV-Vis absorption spectra of **S-tBuCuPc** in THF solutions of 2–12 μM concentration range at room temperature. 14

Fig. S16 UV-Vis absorption spectra of **S-tBuZnPc** in THF solutions of 2–12 μM concentration range at room temperature. 15

Fig. S17 UV-Vis absorption spectra of **S-tBuNiPc** in THF solutions of 2–12 μM concentration range at room temperature. 15

Fig. S18 UV-Vis absorption spectra of **S-$tBuH_2Pc$** in THF solutions of 2–12 μM concentration range at room temperature. 16

Fig. 19 Concentration-dependent absorbance plots of Q-band maxima for the Pcs derivatives in THF at room temperature. (a) *tert*-butyl substituted Pcs (b) S-*tert*-butyl-substituted Pcs.........16

2. Quantum Yield Determination 17

Fig. S20 Fluorescence spectra of tBuZnPc (a), $tBuH_2Pc$ (b), S-tBuZnPc (c), and S-$tBuH_2Pc$ (d) recorded in THF at different concentrations (0.25–1.50 μM) upon excitation at 635 nm........... 18

Fig. S21 Antibiofilm activity of *S. aureus* 19

Fig. S22 Antibiofilm activity of *S. aureus* with PDT activity 20

Fig. S23 Antibiofilm activity of *P. aeruginosa* 20

Fig. S24 Antibiofilm activity *P. aeruginosa* with PDT activity.. 20

Fig. 25 DNA Clevage activity 21

Table 1. Calculated reactivity descriptors for studied chemical systems.................................... 22

1. Synthesis and Characterization of MPcs Derivatives

General procedure for the synthesis of 2(3),9(10),16(17),23(24)-Tetrakis(tert-butyl)metallo-phthalocyanines (tBuZnPc, tBuNiPc, tBuCuPc):

A mixture of tert-butyl-phthalonitril (0.20 g, 1.086 mmol), metal salts (2.16 mmol, 0.396 g Zn $(OAc)_2$, 0.280 g $NiCl_2$ and 0.290 g $CuCl_2$, and a catalytic amount of DBU in n-pentanol (2 mL) was heated at 140 °C with stirring for 24 h under argon. After cooling to room temperature, the reaction mixture was precipitated by adding ethanol. The precipitate was filtered off and washed with ethanol several times.

2(3),9(10),16(17),23(24)-tetrakis(tert-butyl)phthalocyaninatozinc(II) (tBuZnPc): The dark blue precipitate was purified by column chromatography on silica gel using 100:1 and (50:1) DCM:ethanol as eluents. Yield: 75 mg (29%). FT-IR: (cm^{-1}): 3073-3011 (Ar-CH), 2957-2867 (Aliph-CH), 1613 (-C=N-). ^{1}H NMR (DMSO-*d6*): δ, ppm 9.36-8.26 (Ar–H, m, 12H), 1.81 (–CH_3, m, 36H). UV–vis (THF) λmax/nm: 348, 671; MALDI-TOF-MS m/z ($C_{48}H_{48}N_8Zn$) found; 802.074 (Calcd. for [M]+ 802.35).

2(3),9(10),16(17),23(24)-tetrakis(tert-butyl)phthalocyaninatonickel(II) (tBuNiPc): The bluish crude product was purified by column chromatography on silica gel using DCM: ethanol (100:5) as eluent. Yield: 67 mg (30%). FT-IR: (cm^{-1}): 3069-3034 (Ar-CH), 2955-2867 (Aliph-CH), 1617 (-C=N-). ^{1}H NMR ($CDCl_3$): δ, ppm 8.90-7.97 (Ar–H, m, 12H), 1.83 (–CH_3, m, 36H). UV–vis (THF) λmax/nm: 332, 667; MALDI-TOF-MS m/z ($C_{48}H_{48}N_8Ni$) found; 795.091 (Calcd. for [M]+ 795.66).

2(3),9(10),16(17),23(24)-tetrakis(tert-butyl)phthalocyaninatocopper(II) (tBuCuPc): The bluish crude product was purified by column chromatography on silica gel using DCM:ethanol (100:5) as eluent. Yield: 55 mg (25%). FT-IR: (cm^{-1}): 3073-3011 (Ar-CH), 2957-2867 (Aliph-CH), 1613 (-C=N-). UV–vis (THF) λmax/nm: 345, 672; MALDI-TOF-MS m/z ($C_{48}H_{48}N_8Cu$) found; 800.156 (Calcd. for [M]+ 800.51).

Synthesis of 2(3),9(10),16(17),23(24)-Tetrakis(tert-butyl)phthalocyanine (tBuH$_2$Pc):

4-(tert-butyl)phthalonitril (0.20 g, 1.086 mmol) and a catalytic amount of DBU in n-pentanol (2 mL) were heated at 140 °C for 24 h under argon. The mixture was cooled to room temperature and poured into 30 mL of ethanol. Blue-green precipitate was filtered off and washed by ethanol several times. The residue was purified by column chromatography on silica gel using DCM/ethanol 100: 2 as the eluent to afford metal-free phthalocyanine. Yield: 48 mg (24%). FT-IR: (cm^{-1}): 3291, 3081-3019 (Ar-CH), 2956-2861 (Aliph-CH), 1616 (-C=N-). ^{1}H NMR ($CDCl_3$): δ, ppm 9.32-8.30 (Ar–H, m, 12H), 1.86 (–CH_3, m, 36H), -1.69 (N–H, s, 2H). UV–vis (THF) λmax/nm: 341, 660, 696; MALDI-TOF-MS m/z ($C_{48}H_{50}N_8$) found = 738.106 (Calcd. for [M]+ 738.98).

General procedure for the synthesis of 2(3),9(10),16(17),23(24)-Tetrakis(tert-butylthio)metallo-phthalocyanines (S-tBuZnPc, S-tBuNiPc, S-tBuCuPc):

A mixture of 4-(tert-butylthio) phthalonitrile (0.235 g, 1.086 mmol), metal salts (2.16 mmol; 0.396 g $Zn(OAc)_2$, 0.280 g $NiCl_2$, or 0.290 g $CuCl_2$), and a catalytic amount of DBU in *n*-pentanol (2 mL) was heated at 140 °C for 24 h under an argon atmosphere. After cooling to room temperature, the reaction mixture was poured into ethanol to precipitate the product.

2(3),9(10),16(17),23(24)-Tetrakis(tert-butylthio)phthalocyaninatozinc(II) (S-tBuZnPc): The bluish crude product was purified by column chromatography on silica gel using DCM:ethanol (100:10) as eluent. **Yield:** 75 mg (30%). FT-IR: (cm^{-1}): 3073-3002 (Ar-CH), 2961-2865 (Aliph-CH), 1601 (-C=N-). ^{1}H NMR (DMSO-d_6): δ, ppm 9.36-8.26 (Ar–H, m, 12H), 1.81 (–CH_3, m, 36H). UV–vis (THF) λmax/nm: 355, 678; MALDI-TOF-MS m/z ($C_{48}H_{48}N_8S_4Zn$) found; 930.319 (Calcd. for [M]+: 930.59).

2(3),9(10),16(17),23(24)-Tetrakis(tert-butylthio)phthalocyaninatocopper(II) (S-tBuCuPc): The bluish crude product was purified by column chromatography on silica gel using DCM:ethanol (100:10) as eluent. **Yield:** 63 mg (25%). FT-IR: (cm^{-1}): 3073-3007 (Ar-CH), 2961-2864 (Aliph-CH), 1601 (-C=N-). UV–vis (THF) λmax/nm: 350, 679; MALDI-TOF-MS m/z ($C_{48}H_{48}N_8S_4Cu$) found; 928.275 (Calcd. for [M]+: 928.75).

Synthesis of 2(3),9(10),16(17),23(24)-Tetrakis(tert-butylthio)phthalocyanine (S-tBuH$_2$Pc): 4-(tert-butylthio) phthalonitrile (0.235 g, 1.086 mmol) and a catalytic amount of DBU were dissolved

in *n*-pentanol (2 mL) and heated at 140 °C for 24 h under an argon atmosphere. After completion, the reaction mixture was cooled to room temperature and poured into 30 mL of ethanol. The resulting blue-green solid was filtered and washed several times with ethanol. The crude product was purified by column chromatography on silica gel using DCM/ethanol (100:2, v/v) as the eluent to afford as a pure product. Yield: 75 mg (31%). FT-IR: (cm^{-1}): 3288 (-NH), 3069-3001 (Ar-CH), 2960-2860 (Aliph-CH), 1603 (-C=N-). ^{1}H NMR ($CDCl_3$): δ, ppm 8.10-7.46 (Ar–H, m, 12H), 1.73-1.63 (–CH_3, m, 36H). –4.44 (N–H, s, 2H). UV–vis (THF) λmax/nm: 343,667,702; MALDI-TOF-MS m/z ($C_{48}H_{50}N_8S_4$) found; 867.024 (Calcd. for [M]+: 867.22).

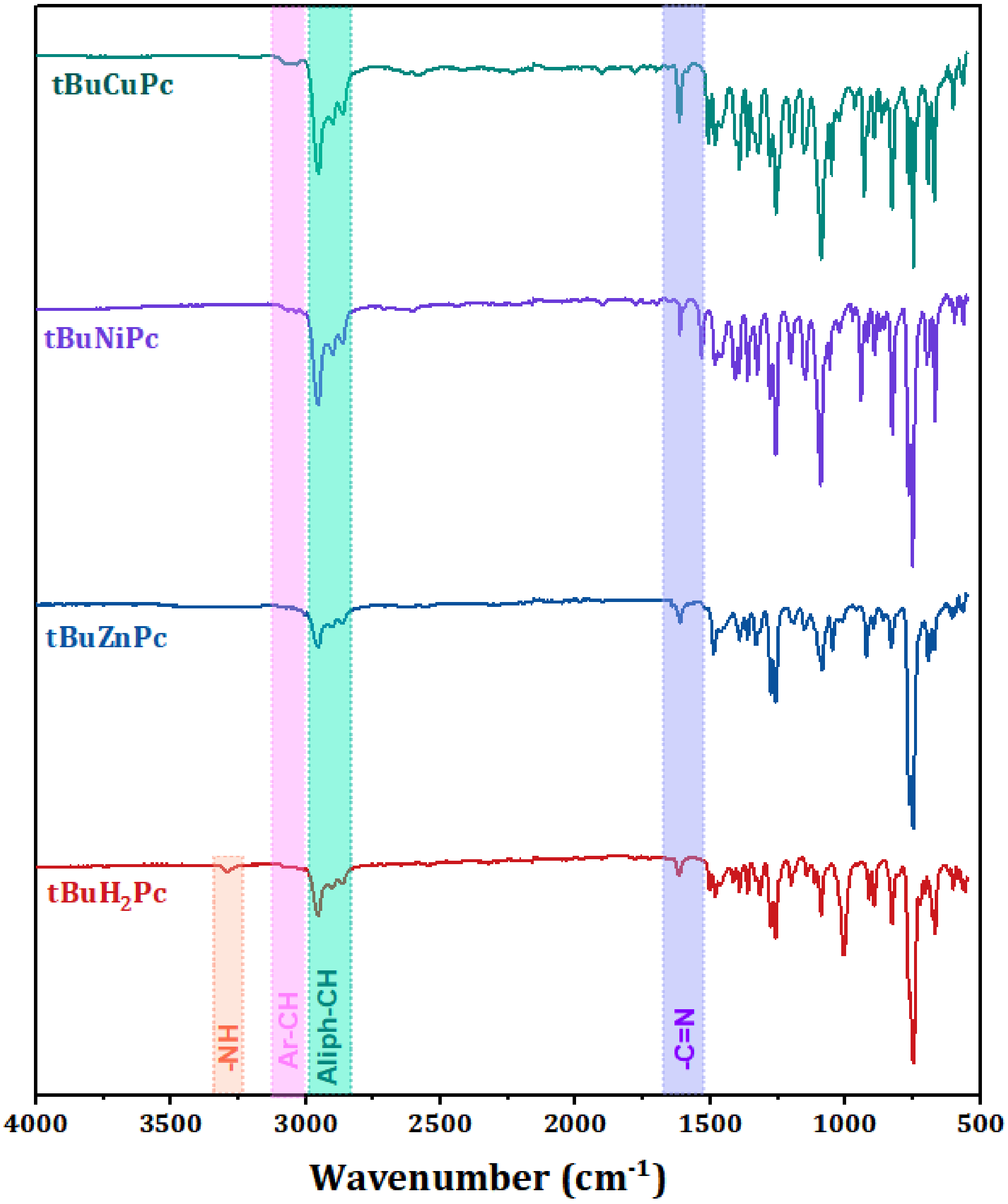

Fig. S1 FT-IR spectra of all **tBuMPc** derivatives.

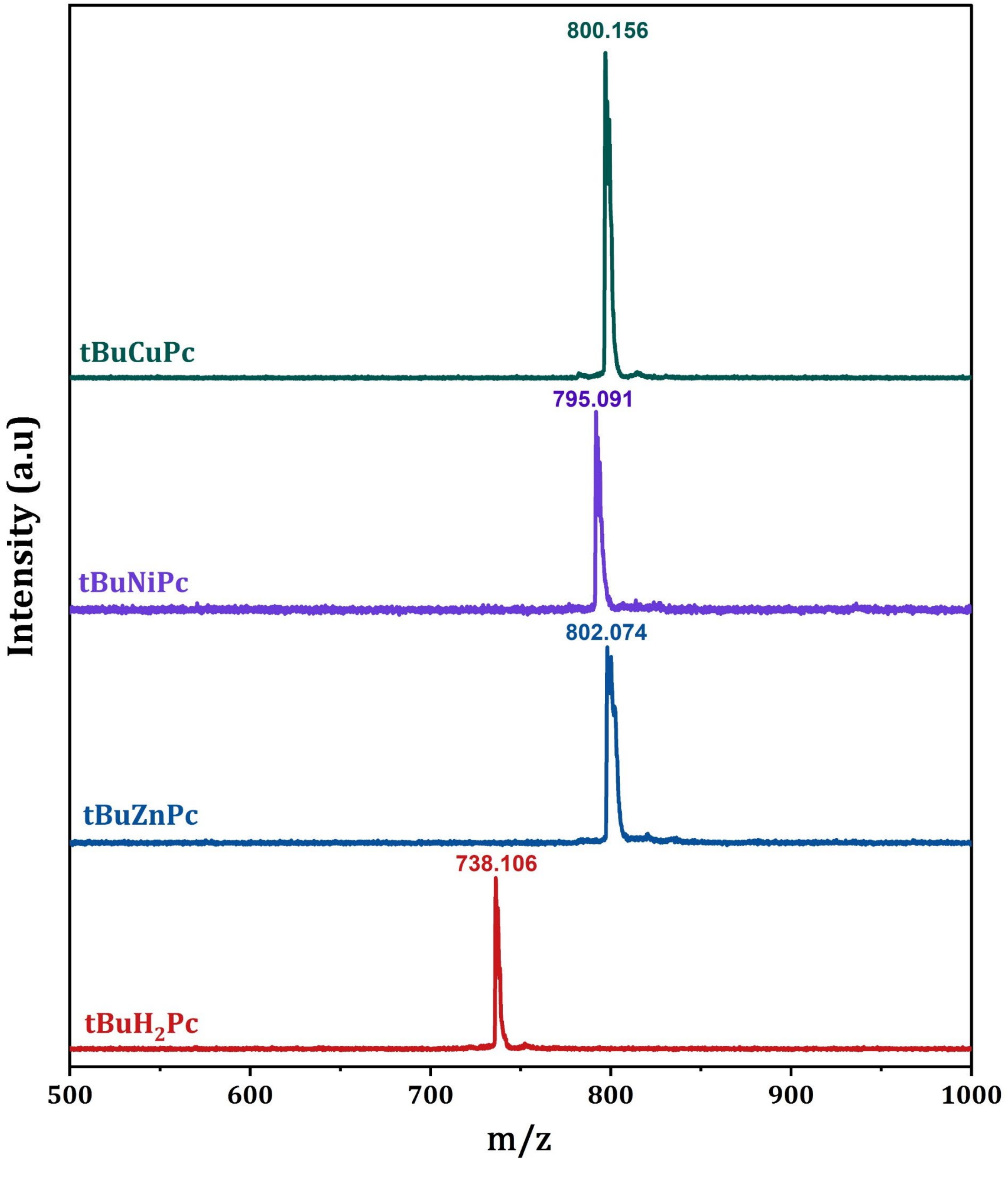

Fig. S2 MALDI-TOF MS spectra of all **tBuMPc** derivatives obtained using the **DIT** matrix**,** with the $[M]^{+\cdot}$ molecular ion peaks marked on the spectra.

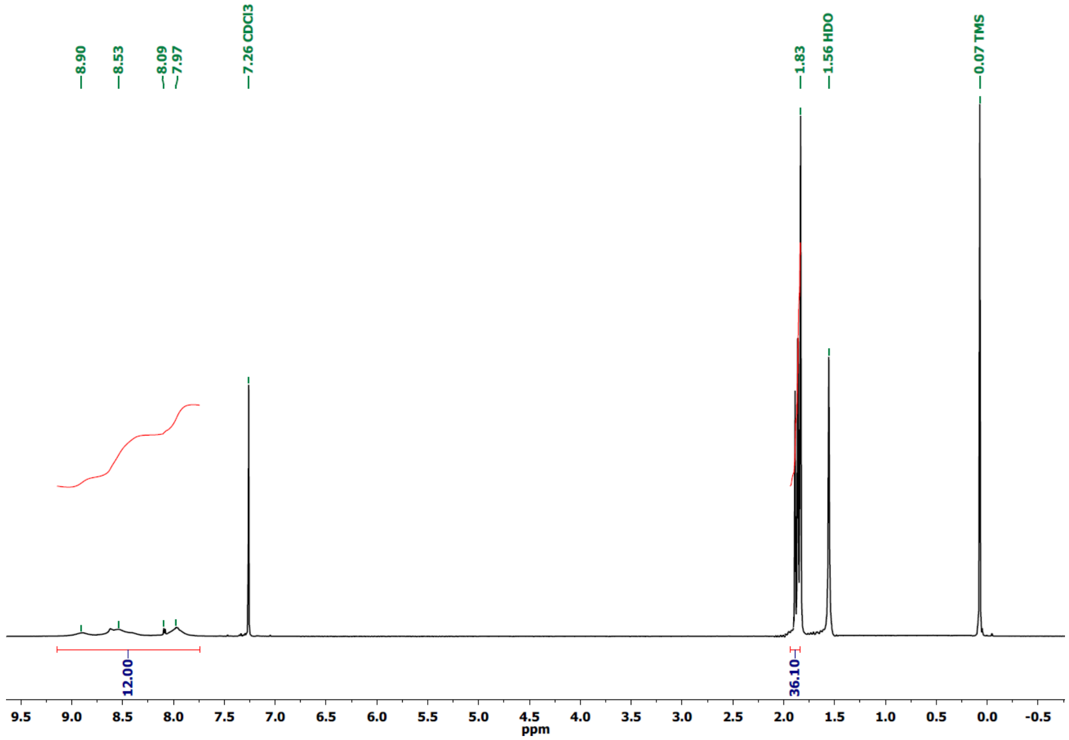

Fig. S3 ^1H NMR spectra for **tBuNiPc** in $CDCl_3$.

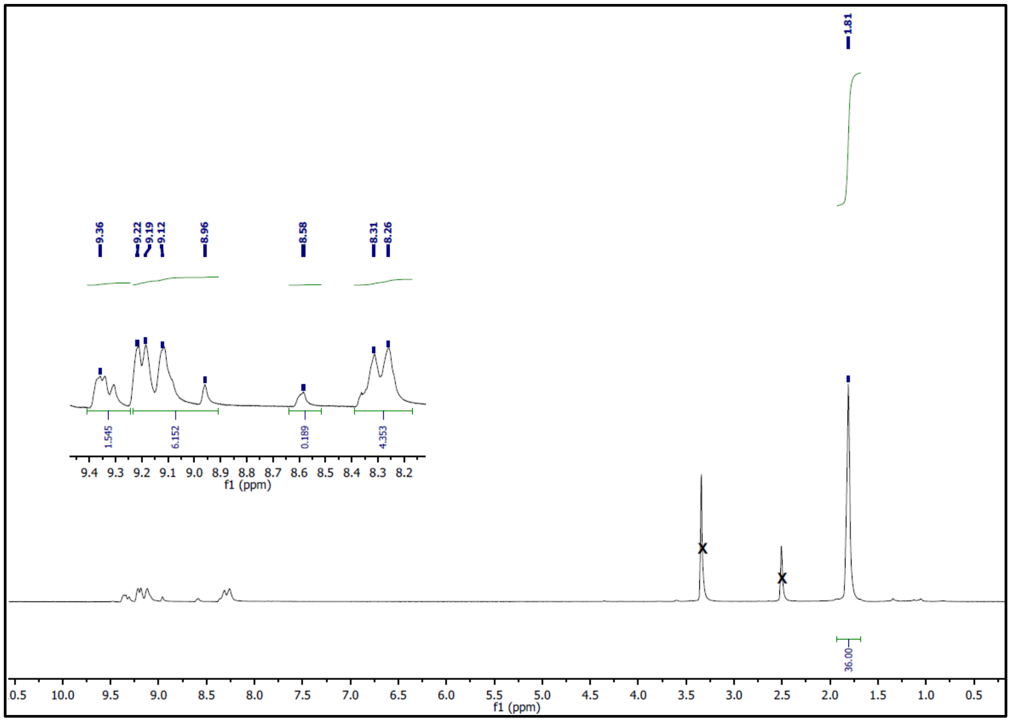

Fig. S4 ^1H NMR spectra for **tBuZnPc** in DMSO-d_6.

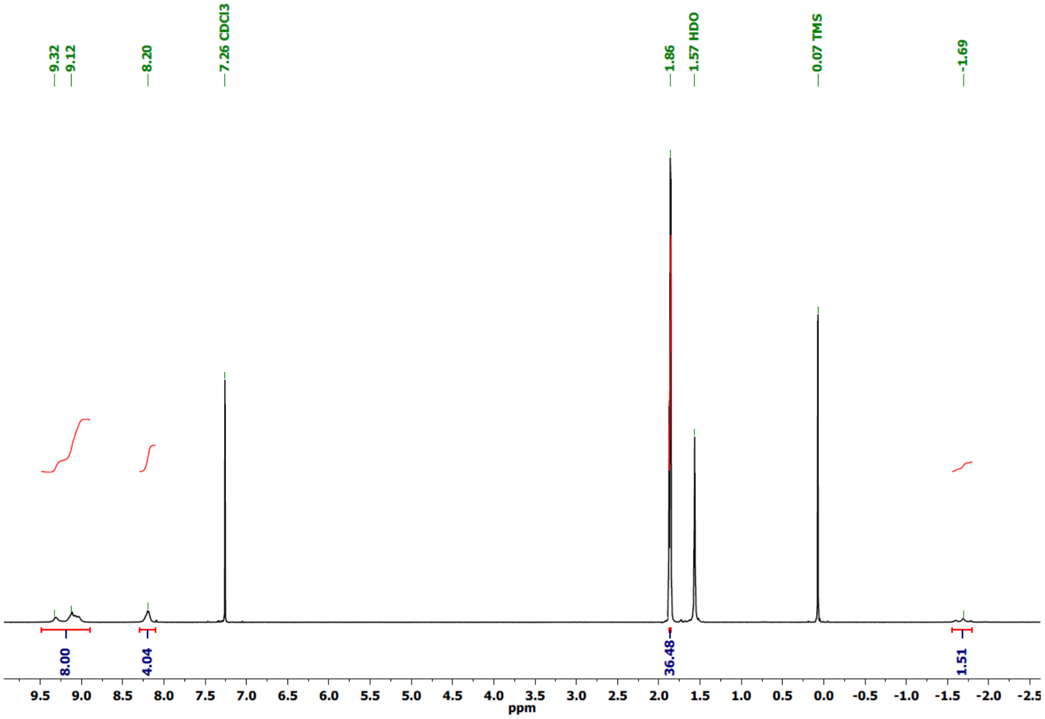

Fig. S5 ^{1}H NMR spectra for **tBuH_2Pc** in $CDCl_3$.

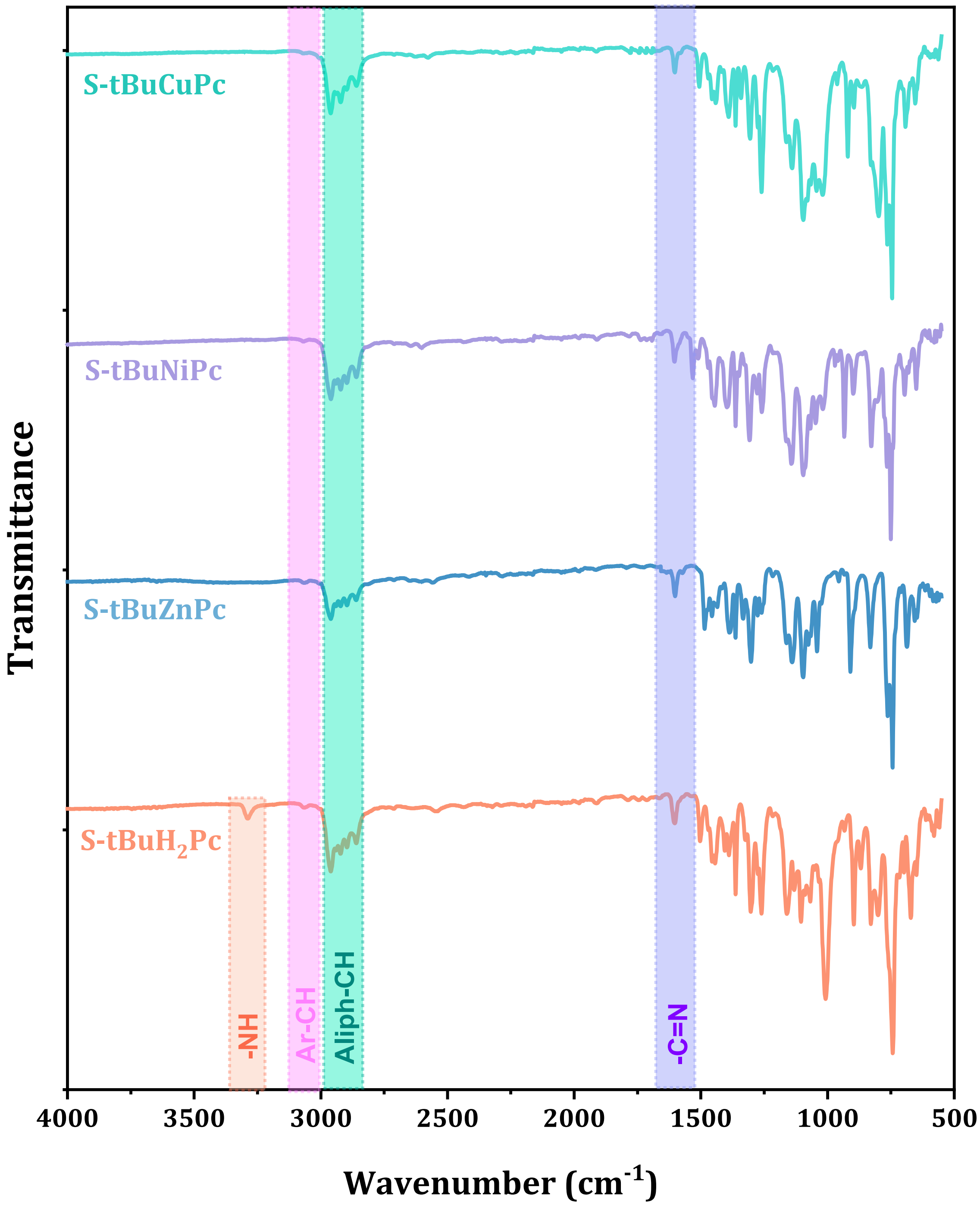

Fig. S6 FT-IR spectra of all **S-tBuMPc** derivatives.

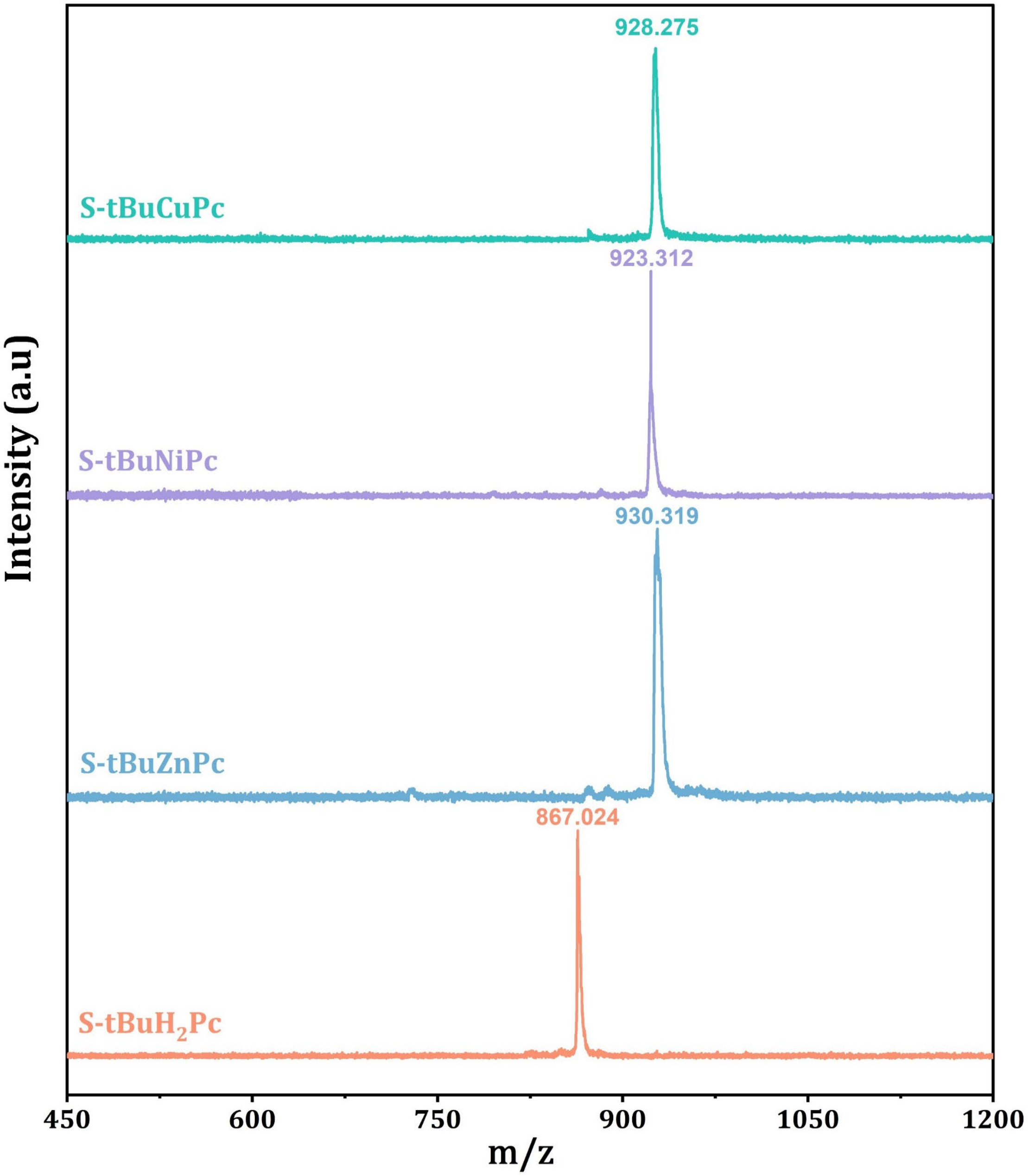

Fig. S7 MALDI-TOF MS spectra of all **S-tBuMPc** derivatives obtained using the **DIT** matrix, with the $[M]^{+\cdot}$ molecular ion peaks marked on the spectra.

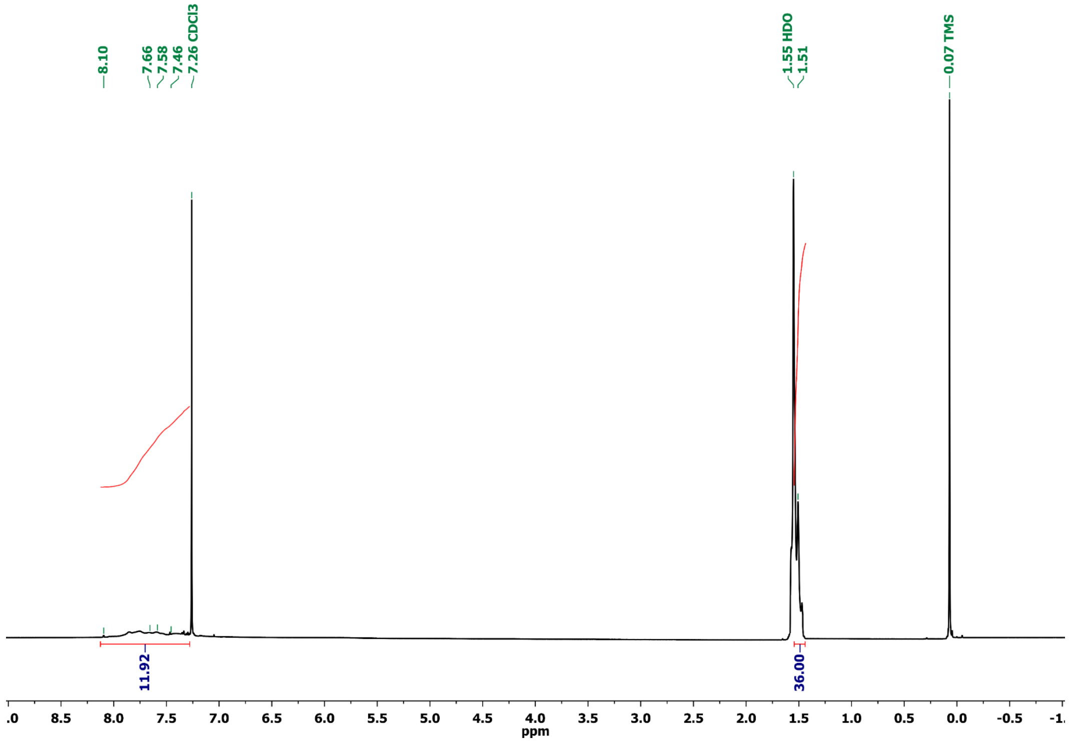

Fig. S8 ^{1}H NMR spectra for **S-tBuNiPc** in $CDCl_3$.

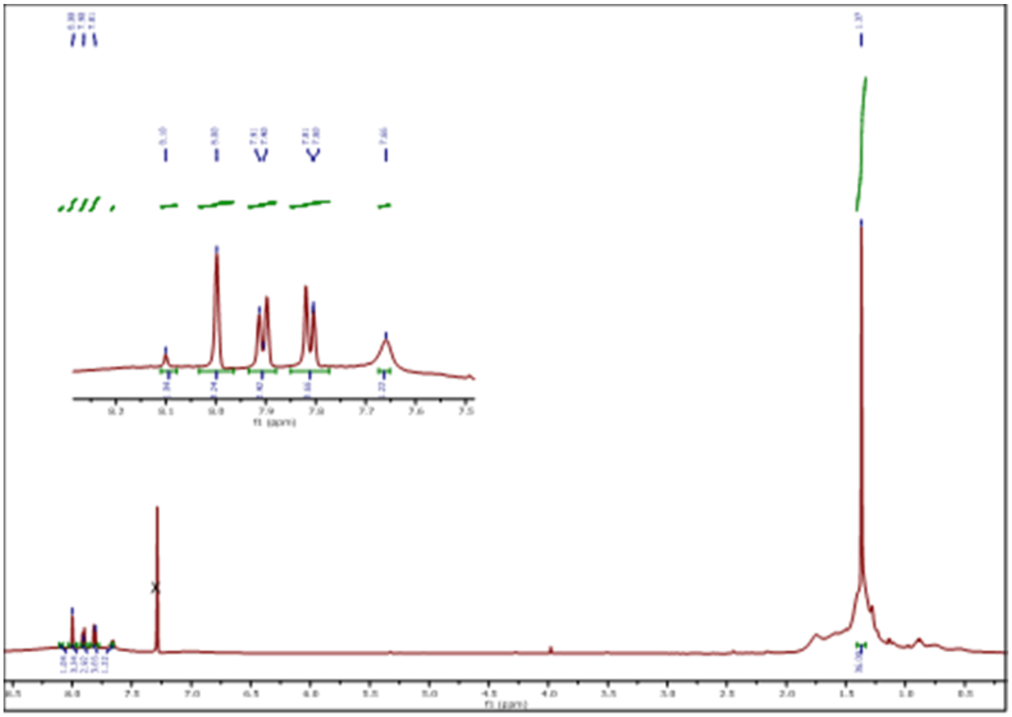

Fig. S9 ^{1}H NMR spectra for **S-tBuZnPc** in $CDCl_3$.

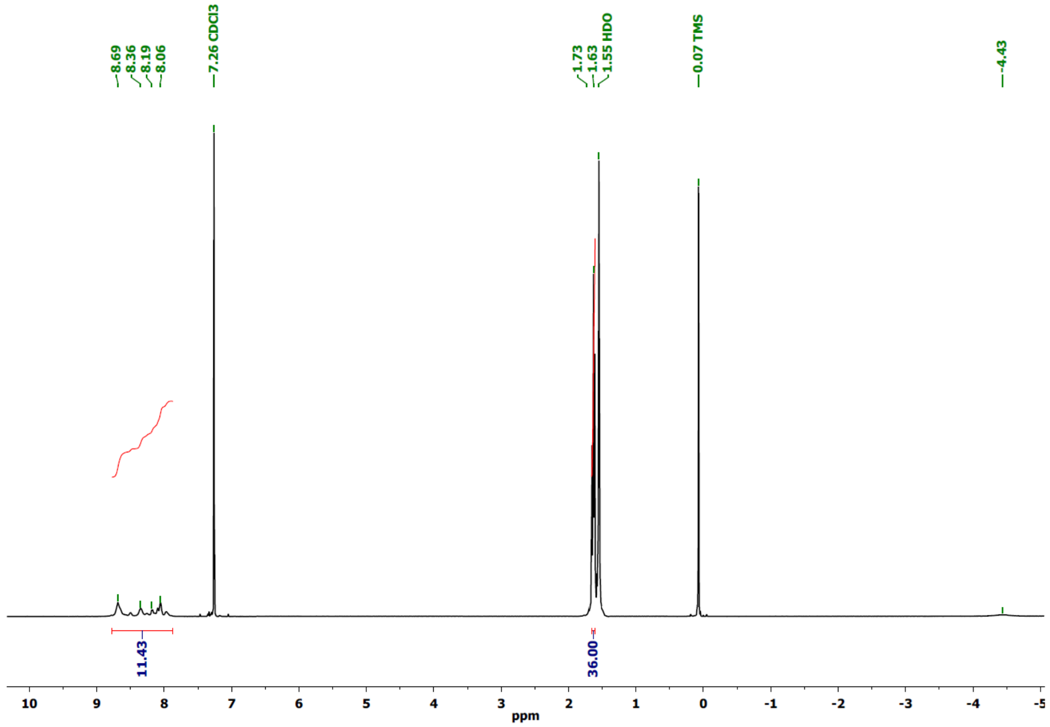

Fig. S10 ^{1}H NMR spectra for **tBuH$_2$Pc** in $CDCl_3$.

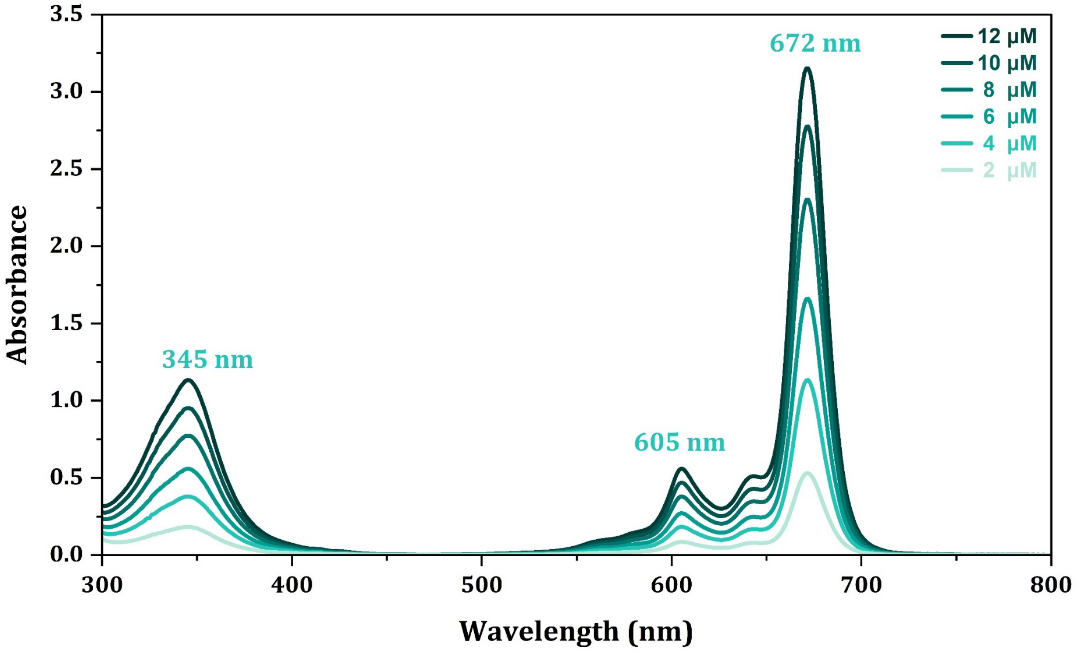

Fig. S11 UV-Vis absorption spectra of **tBuCuPc** in THF solutions of 2–12 μM concentration range at room temperature.

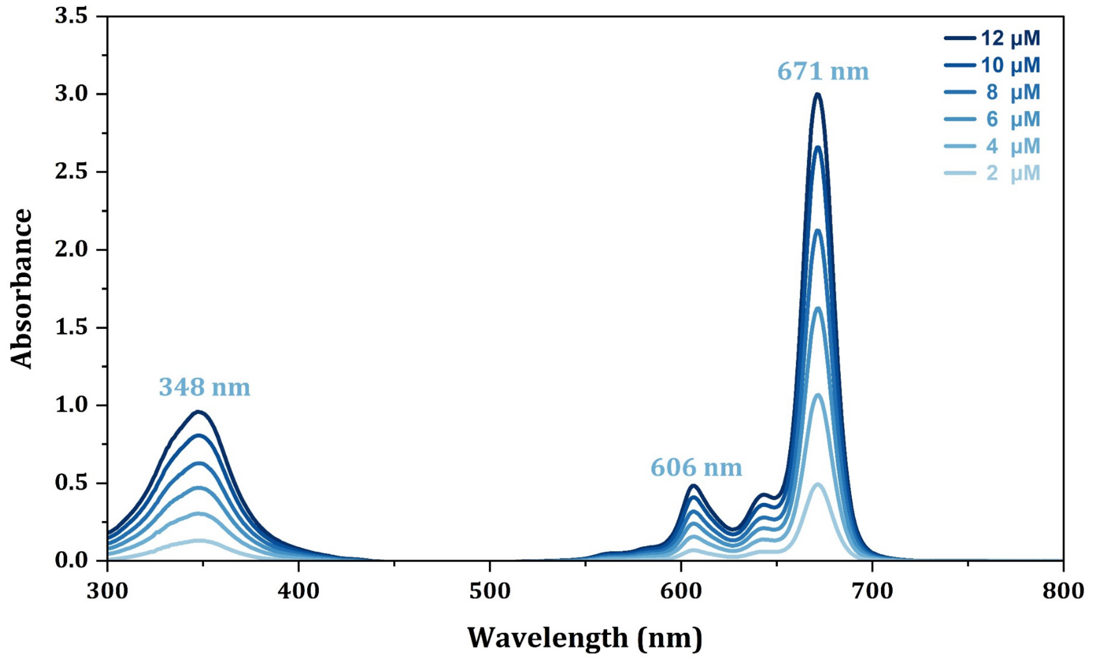

Fig. S12 UV-Vis absorption spectra of **tBuZnPc** in THF solutions of 2–12 μM concentration range at room temperature.

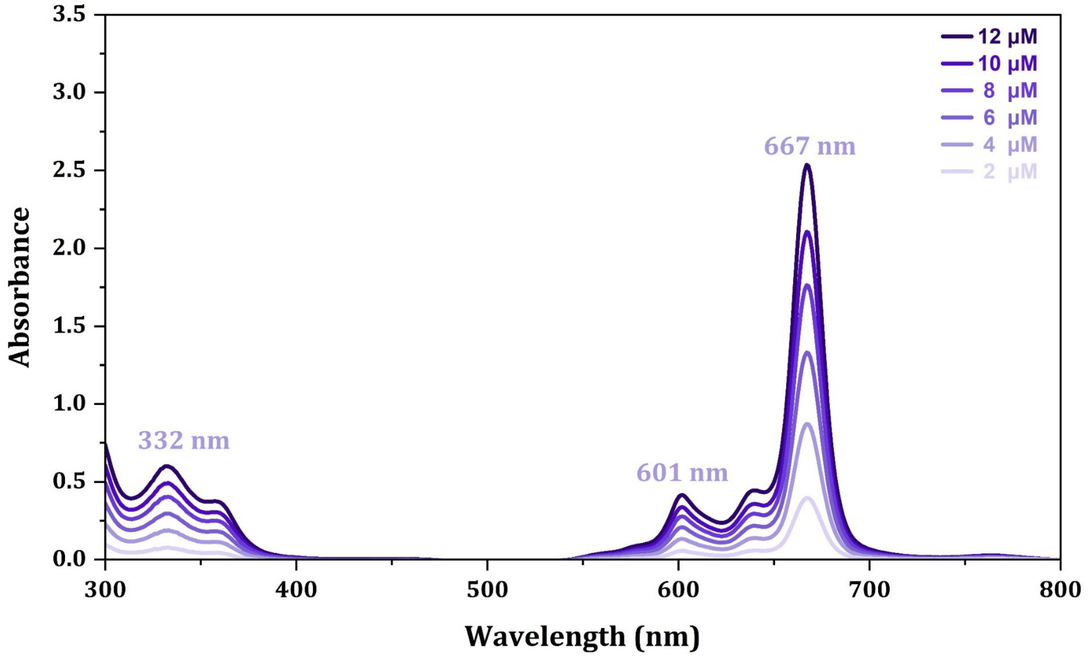

Fig. S13 UV-Vis absorption spectra of **tBuNiPc** in THF solutions of 2–12 μM concentration range at room temperature.

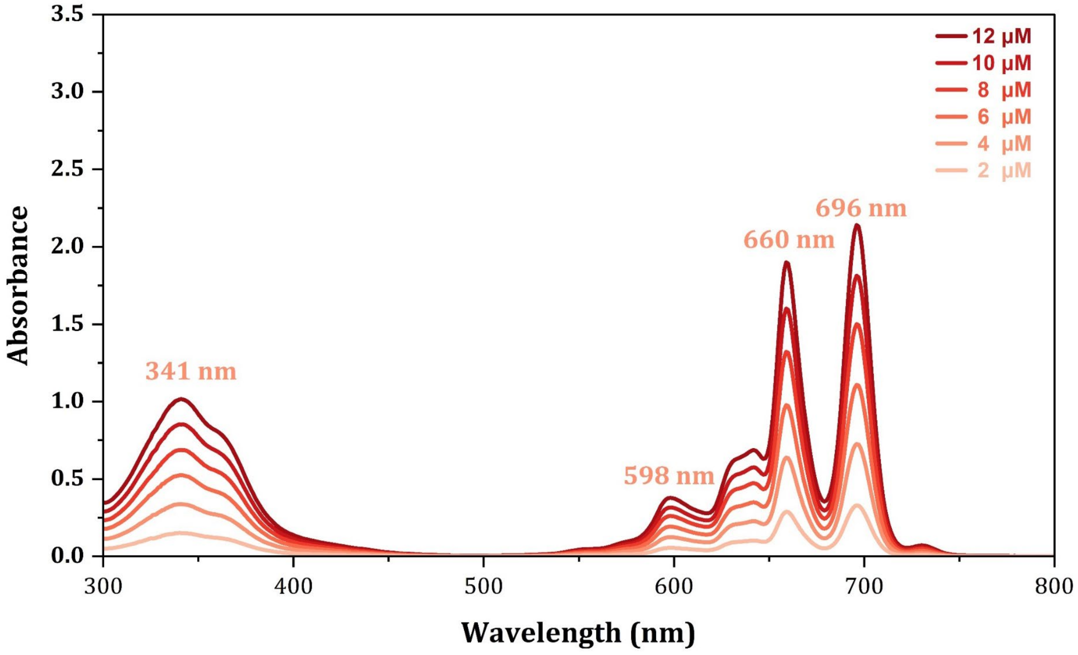

Fig. S14 UV-Vis absorption spectra of **tBuH$_2$Pc** in THF solutions of 2–12 μM concentration range at room temperature.

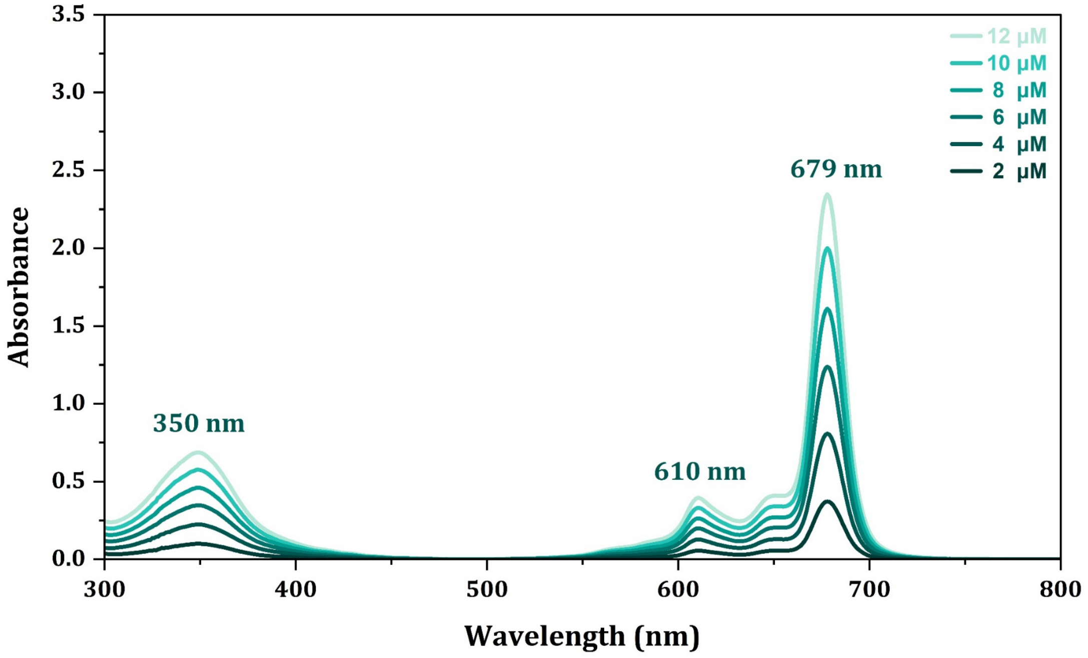

Fig. S15 UV-Vis absorption spectra of **S-tBuCuPc** in THF solutions of 2–12 μM concentration range at room temperature.

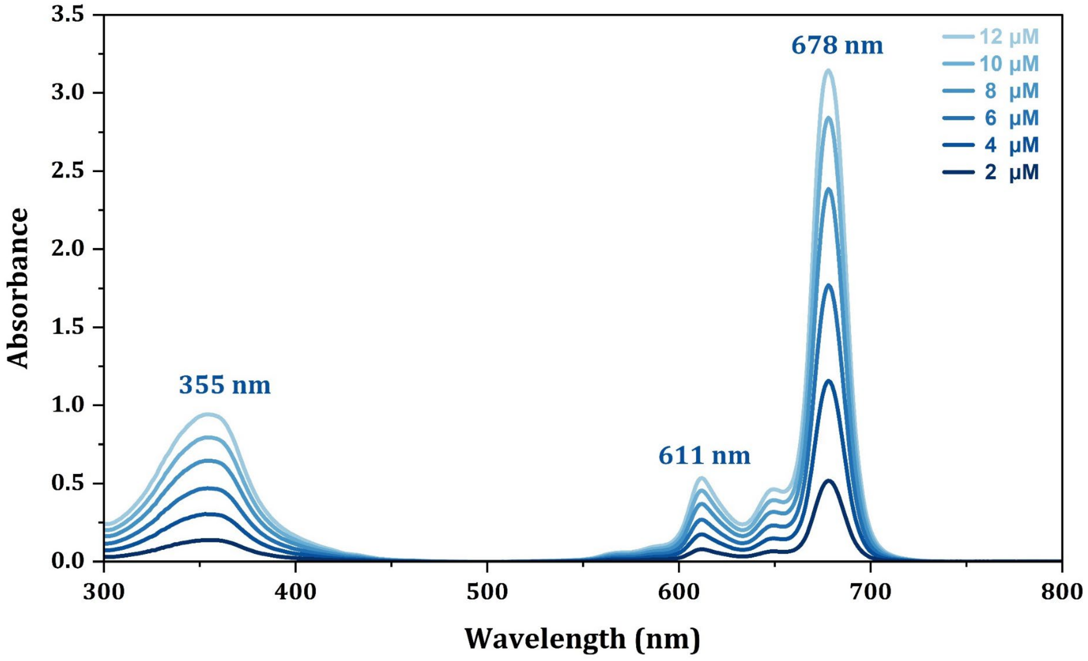

Fig. S16 UV-Vis absorption spectra of **S-tBuZnPc** in THF solutions of 2–12 μM concentration range at room temperature.

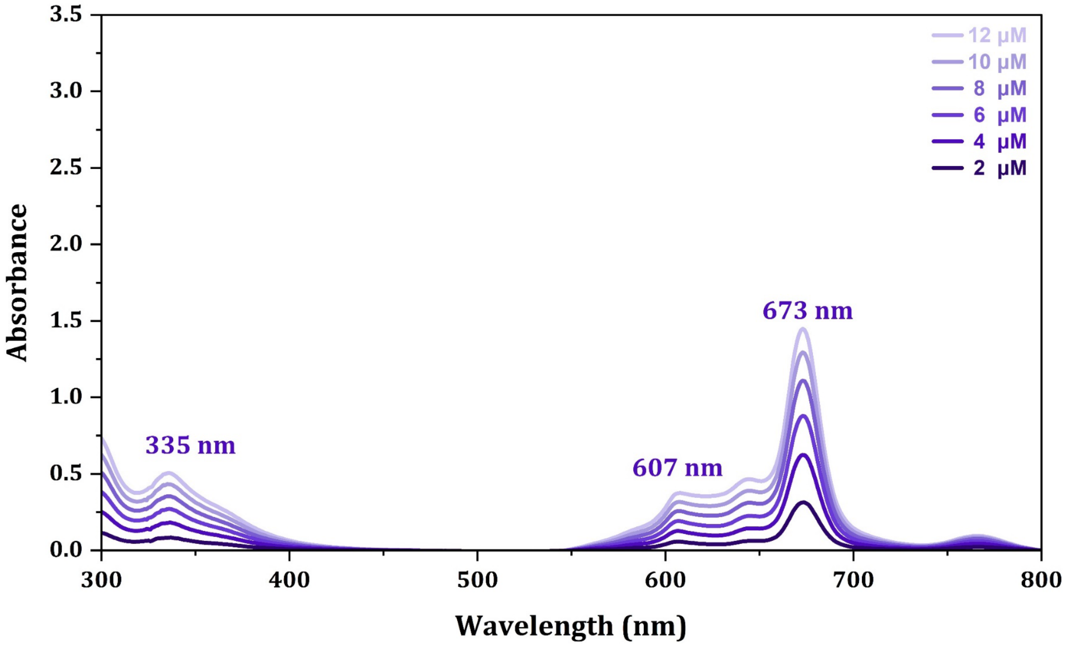

Fig. S17 UV-Vis absorption spectra of **S-tBuNiPc** in THF solutions of 2–12 μM concentration range at room temperature.

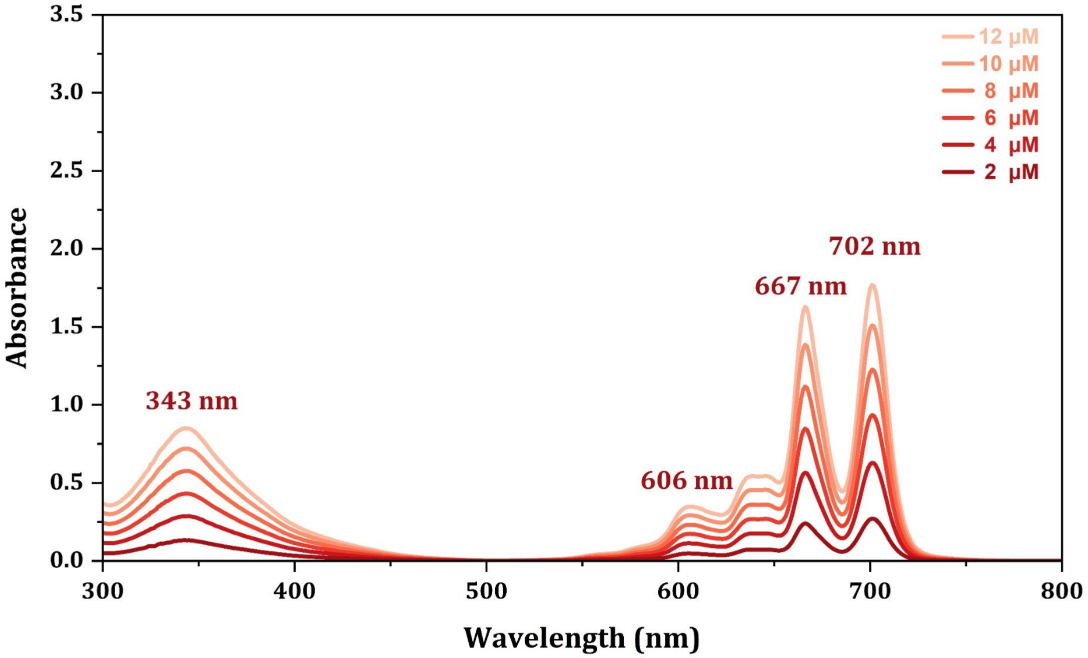

Fig. S18 UV-Vis absorption spectra of **S-tBuH₂Pc** in THF solutions of 2–12 μM concentration range at room temperature.

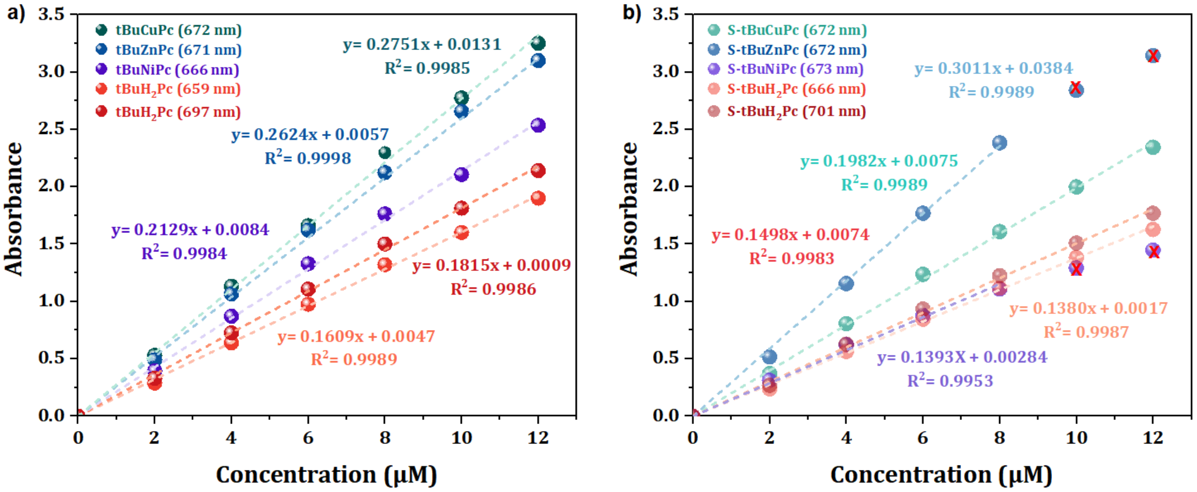

Fig. 19 Concentration-dependent absorbance plots of Q-band maxima for the Pcs derivatives in THF at room temperature. (a) *tert*-butyl substituted Pcs (b) S-*tert*-butyl-substituted Pcs.

2. Quantum Yield Determination

The fluorescence quantum yields (Φ_F) of the MPcs were determined using the relative method. https://doi.org/10.1088/2050-6120/ab7e10 In this approach, the quantum yield of the sample is calculated by comparison with a standard of known quantum yield (Φ_F^{std}) under the same experimental conditions. The calculations were performed using the following equations (Eq 1-3).

$$\Phi_\mathrm{F} = \Phi_F^{std} \left(\frac{I_S}{I_{std}}\right) \left(\frac{1-10^{-A_{std}}}{1-10^{-A_S}}\right) \left(\frac{\eta_S}{\eta_{std}}\right)^2 \qquad \text{Eq.1}$$

$$\Phi_\mathrm{F} = \Phi_R \left(\frac{I_{std}}{1-10^{-A_S}}\right) \left(\frac{1-10^{-A_{std}}}{I_{A_S}}\right) \left(\frac{\eta_S}{\eta_{std}}\right)^2 \qquad \text{Eq.2}$$

$$\Phi_\mathrm{F} = \Phi_F^{std} \left(\frac{Grad_S}{Grad_{std}}\right) \left(\frac{\eta_S}{\eta_{std}}\right)^2 \qquad \text{Eq.3}$$

The equations, I represent the integrated fluorescence intensity, A is the absorbance at the excitation wavelength, and η is the refractive index of the solvent. *Grad* denotes the slope obtained from the plot of integrated fluorescence intensity versus $1 - 10^{-A_s\ or\ A_{std}}$.

In this study, unsubstituted ZnPc (Φ_F^{std} =0.30 in THF) was used as the reference fluorophore. Solutions of std-ZnPc and the MPcs were prepared in THF within the concentration range of 0.5–2.0 µM. The obtained spectra are presented in Figure S21. Then, their absorbance and fluorescence spectra were recorded under the same experimental conditions. The integrated emission intensities were plotted against $1 - 10^{-A_s\ or\ A_{std}}$ to obtain linear correlations, and the slopes (Grads) were used to calculate the relative Φ_F values according to Eq. 3. The corresponding linear correlations used for quantum yield determination are given in Figure S22.

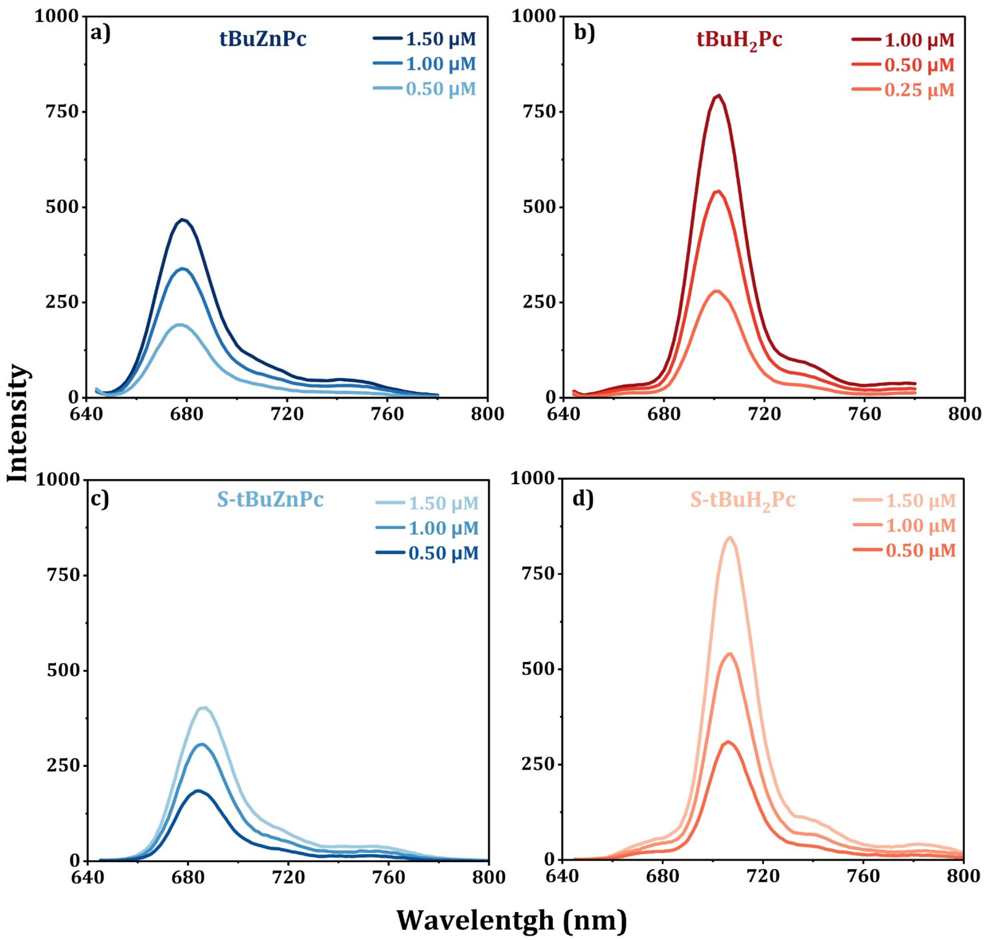

Fig. S19 Fluorescence spectra of tBuZnPc (a), $tBuH_2Pc$ (b), S-tBuZnPc (c), and S-$tBuH_2Pc$ (d) recorded in THF at different concentrations (0.25–1.50 μM) upon excitation at 635 nm.

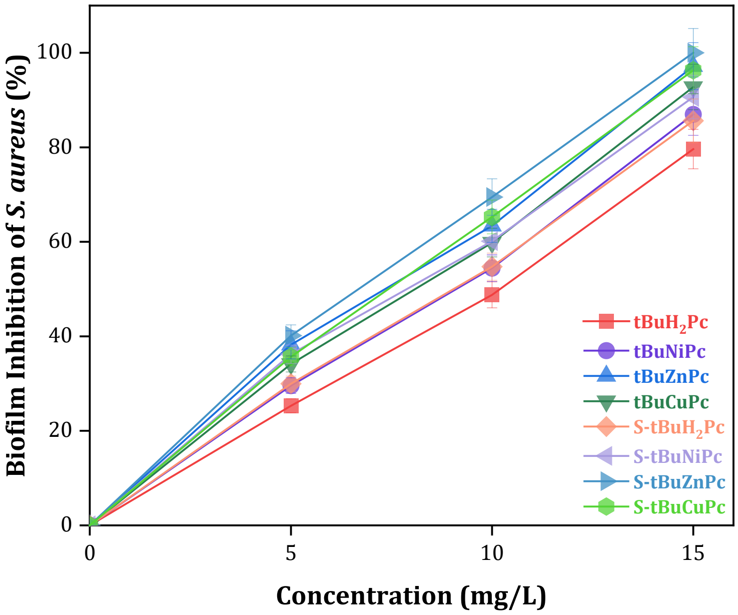

Fig. S20 Antibiofilm activity of *S. aureus*

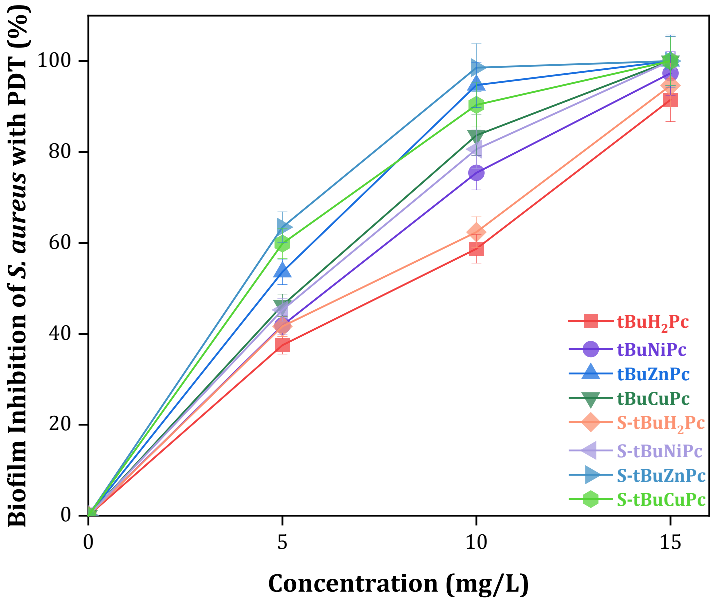

Fig. S21 Antibiofilm activity of *S. aureus* with PDT activity

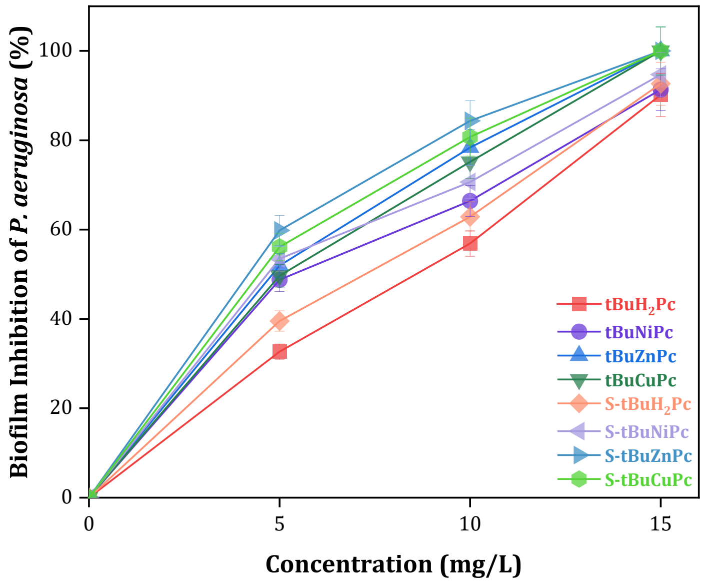

Fig. S22 Antibiofilm activity of *P. aeruginosa*

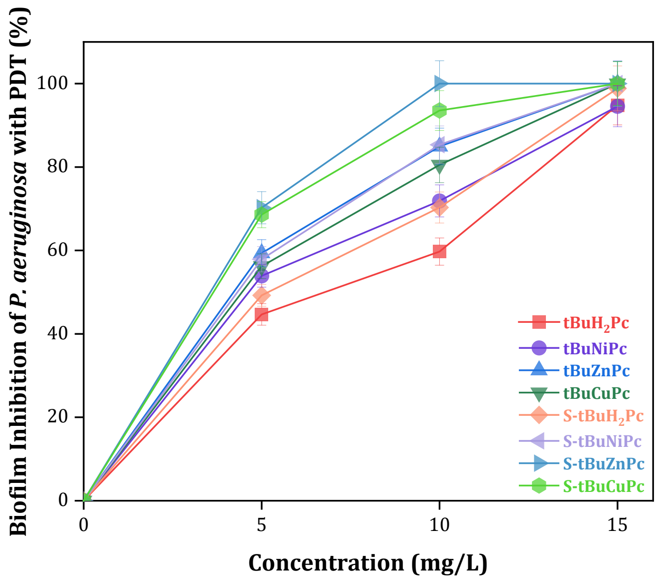

Fig. S23 Antibiofilm activity *P. aeruginosa* with PDT activity

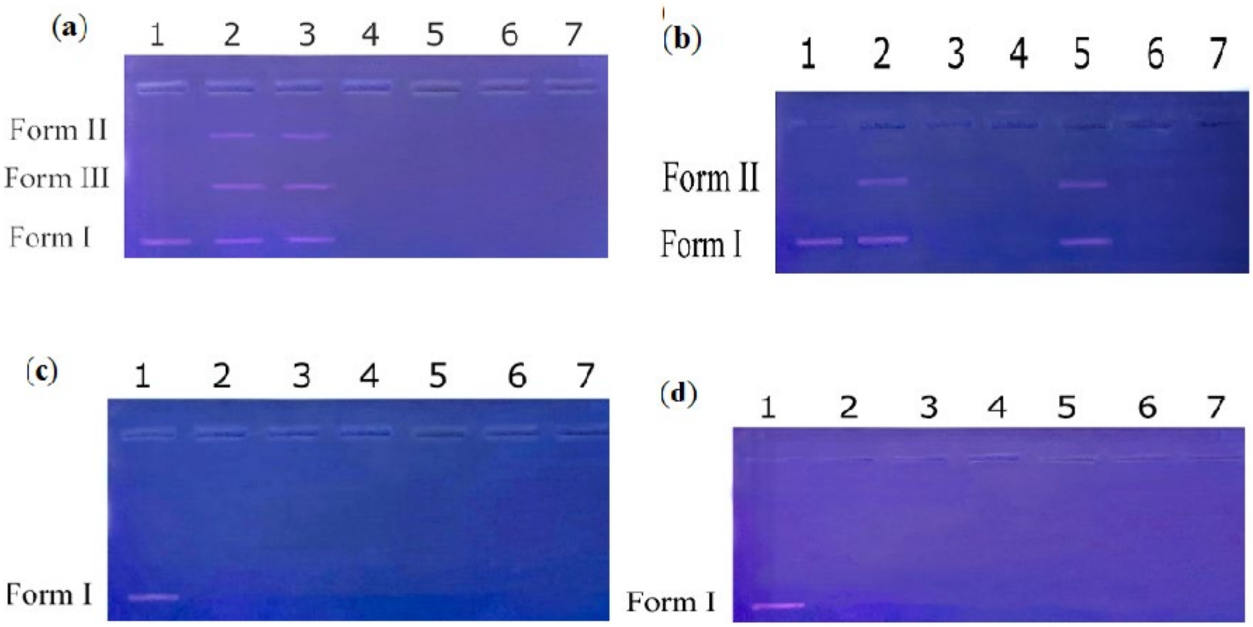

Fig. 24 DNA Clevage activity. **(a)** 1: DMSO + pBR 322 DNA; 2: 50 mg/L of **tBuH$_2$Pc** + pBR 322 DNA; 3: 100 mg/L of **tBuH$_2$Pc** +pBR 322 DNA; 4: 200 mg/L of **tBuH$_2$Pc** +pBR 322 DNA; 5: 50 mg/L of **tBuNiPc** +pBR 322 DNA; 6: 100 mg/L of **tBuNiPc** + pBR 322 DNA; 7: 200 mg/L of **tBuNiPc 2**+pBR 322 DNA; **(b)** 1: DMSO+pBR 322 DNA; 2: 50 mg/L of **tBuZnPc** +pBR 322 DNA; 3: 100 mg/L of **tBuZnPc** +pBR 322 DNA; 4: 200 mg/L of **tBuZnPc** +pBR 322 DNA; 5: 50 mg/L of **tBuCuPc** +pBR 322 DNA; 6: 100 mg/L of **tBuCuPc** + pBR 322 DNA; 7: 200 mg/L of **tBuCuPc** + pBR 322 DNA; **(c)** 1: DMSO + pBR 322 DNA; 2: 50 mg/L of **S-tBuH$_2$Pc** + pBR 322 DNA; 3: 100 mg/L of **S-tBuH$_2$Pc** + pBR 322 DNA; 4: 200 mg/L of **S-tBuH$_2$Pc** + pBR 322 DNA; 5: 50 mg/L of **S-tBuNiPc** + pBR 322 DNA; 6: 100 mg/L of **S-tBuNiPc** + pBR 322 DNA; 7: 200 mg/L of **S-tBuNiPc** + pBR 322 DNA; **(d)** 1: DMSO+pBR 322 DNA; 2: 50 mg/L of **S-tBuZnPc** + pBR 322 DNA; 3: 100 mg/L of **S-tBuZnPc** + pBR 322 DNA; 4: 200 mg/L of **S-tBuZnPc** + pBR 322 DNA; 5: 50 mg/L of **S-tBuCuPc** + pBR 322 DNA; 6: 100 mg/L of **S-tBuCuPc** + pBR 322 DNA; 7: 200 mg/L of **S-tBuCuPc** + pBR 322 DNA.

Compound	Energy [Ha]	E_{HOMO} (eV)	E_{LUMO} (eV)	χ	H	$ω_1$	$ω_2$
**tBuH$_2$Pc**	-2297.6259081	-4.846	-3.421	4.134	1.425	5.995	11.634
**tBuCuPc**	-3937.0895228	-4.479	-3.535	4.007	0.944	8.504	16.773
**tBuNiPc**	-3804.9428631	-5.009	-3.508	4.259	1.501	6.041	11.707
**tBuZnPc**	-4075.9304784	-4.984	-3.528	4.256	1.456	6.220	12.077
**S-tBuH$_2$Pc**	-3890.4723549	-4.811	-3.523	4.167	1.288	6.741	13.159
**S-tBuCuPc**	-5529.8774793	-4.481	-3.607	4.044	0.874	9.356	18.493
**S-tBuNiPc**	-5397.7299042	-4.954	-3.566	4.260	1.388	6.537	12.728
**S-tBuZnPc**	-5668.7199837	-5.035	-3.628	4.332	1.407	6.667	12.983

Table S1. Calculated reactivity descriptors for studied chemical systems.
